# Epigenetic control of cellular crosstalk defines gastrointestinal organ fate and function

**DOI:** 10.1038/s41467-023-36228-2

**Published:** 2023-01-30

**Authors:** Ryan J. Smith, Minggao Liang, Adrian Kwan Ho Loe, Theodora Yung, Ji-Eun Kim, Matthew Hudson, Michael D. Wilson, Tae-Hee Kim

**Affiliations:** 1 Program in Developmental & Stem Cell Biology, The Hospital for Sick Children, Toronto, ON M5G 0A4 Canada; 2 Department of Molecular Genetics, University of Toronto, Toronto, ON M5S 1A8 Canada; 3 Program in Genetics and Genome Biology, The Hospital for Sick Children, Toronto, ON M5G 0A4 Canada

**Keywords:** Stem-cell niche, Stem-cell niche, RNA sequencing, Chromatin

## Abstract

Epithelial-mesenchymal signaling in the gastrointestinal system is vital in establishing regional identity during organogenesis and maintaining adult stem cell homeostasis. Although recent work has demonstrated that Wnt ligands expressed by mesenchymal cells are required during gastrointestinal development and stem cell homeostasis, epigenetic mechanisms driving spatiotemporal control of crosstalk remain unknown. Here, we demonstrate that gastrointestinal mesenchymal cells control epithelial fate and function through Polycomb Repressive Complex 2-mediated chromatin bivalency. We find that while key lineage-determining genes possess tissue-specific chromatin accessibility, Polycomb Repressive Complex 2 controls Wnt expression in mesenchymal cells without altering accessibility. We show that reduction of mesenchymal Wnt secretion rescues gastrointestinal fate and proliferation defects caused by Polycomb Repressive Complex 2 loss. We demonstrate that mesenchymal Polycomb Repressive Complex 2 also regulates niche signals to maintain stem cell function in the adult intestine. Our results highlight a broadly permissive chromatin architecture underlying regionalization in mesenchymal cells, then demonstrate further how chromatin architecture in niches can influence the fate and function of neighboring cells.

## Abstract (web summary)

Mesenchymal-epithelial crosstalk plays a key role in gut development and stem cell homeostasis, though underlying mechanisms are still unclear. Here, the authors demonstrate that mesenchymal Polycomb Repressive Complex 2 controls niche signals for gut epithelial fate and growth.

## Introduction

Although intrinsic epigenetic mechanisms of organ specification and stem cell differentiation have been studied extensively^1^, little is known about epigenetic regulation of tissue-cross talk. The gastrointestinal (GI) system is a well characterized model to study intercellular communication. It is known that mesenchymal signals control organ specification and growth, as well as stem cell renewal and differentiation in GI development and adult homeostasis, respectively^2,3^.

In early GI development, cross-sections of the putative gastric and intestinal regions reveal a ring of undifferentiated mesenchymal cells surrounding a single-cell layer of pseudostratified epithelial cells^2^. Despite these morphological similarities, regional differences in gene expression demarcate organ boundaries. A few organ- and region-specific mesenchymal homeobox transcription factors (TFs) have been known to play critical roles in GI organ specification and regional identity. *Hox* genes display regionalized expression patterns along the proximal-distal axis: 3′ genes are expressed in proximal regions, whereas 5′ genes are restricted to distal regions^4^. While loss of function studies for individual *Hox* genes have shown mild or no obvious defects, suggesting redundancy due to their significantly overlapping expression patterns^5^, gain of function studies for distal *Hox* genes in the chick midgut mesenchyme have demonstrated their capacity to differentiate the overlying epithelium toward a distal gut identity^6^. The expression of the mesenchyme-specific TF *Barx1* denotes the prospective gastric region, and its loss leads to a loss of gastric fate; Wnt activity is low in the developing stomach, with BARX1 activating Wnt antagonists such as the secreted Frizzled-related proteins, sFRP1 and sFRP2^7^. While these studies highlight how mesenchymal TFs influence GI organ specification and regional identity through the regulation of niche signals, the epigenetic mechanisms underlying this tissue crosstalk are currently unknown.

Wnt signaling is essential for intestinal development and stem cells^8^. Paneth cells surrounding Lgr5+ intestinal stem cells are a critical source of Wnt ligands for stem cell activity and organoid formation^9^. Interestingly, upon depletion of Paneth cells in vivo, stem cell activity remains intact, suggesting additional sources of Wnt ligands^10,11^. Corroborating this data, epithelial Wnt ligand knockout and genetic inhibition of Wnt ligand secretion does not lead to obvious defects in the intestine^12–14^. Mesenchymal cells express various Wnt ligands in both GI development and adult homeostasis^15–18^. Indeed, the genetic inhibition of Wnt secretion from pericryptal mesenchymal cells in close proximity to gut epithelial stem cells, and mesenchymal cells more broadly leads to stem cell and GI developmental defects, respectively, demonstrating the importance of mesenchymal factors for GI organ development and stem cell functionality^19–24^. However, it remains unclear how these mesenchymal niche signals are controlled.

Here, we sought to identify the role of chromatin in regulating the intercellular communication critical to proper development of the gastrointestinal system. Utilizing gastrointestinal mesenchyme-specific reporter mice, we analyzed mesenchymal chromatin patterns and gene expression. Although key lineage-defining genes such as *Barx1* and *Hox* genes undergo tissue-specific chromatin regulation, we surprisingly found broadly permissive and highly similar chromatin accessibility patterns in both stomach and intestinal mesenchymal cells. By genetically inhibiting the Polycomb Repressive Complex 2 (PRC2), we have demonstrated that PRC2 controls mesenchymal niche signals essential for GI organ specification and proliferation through the maintenance of bivalent gene promoters.

## Results

### Gastrointestinal mesenchymal cells possess broadly similar chromatin accessibility profiles

**Fig. 1:**
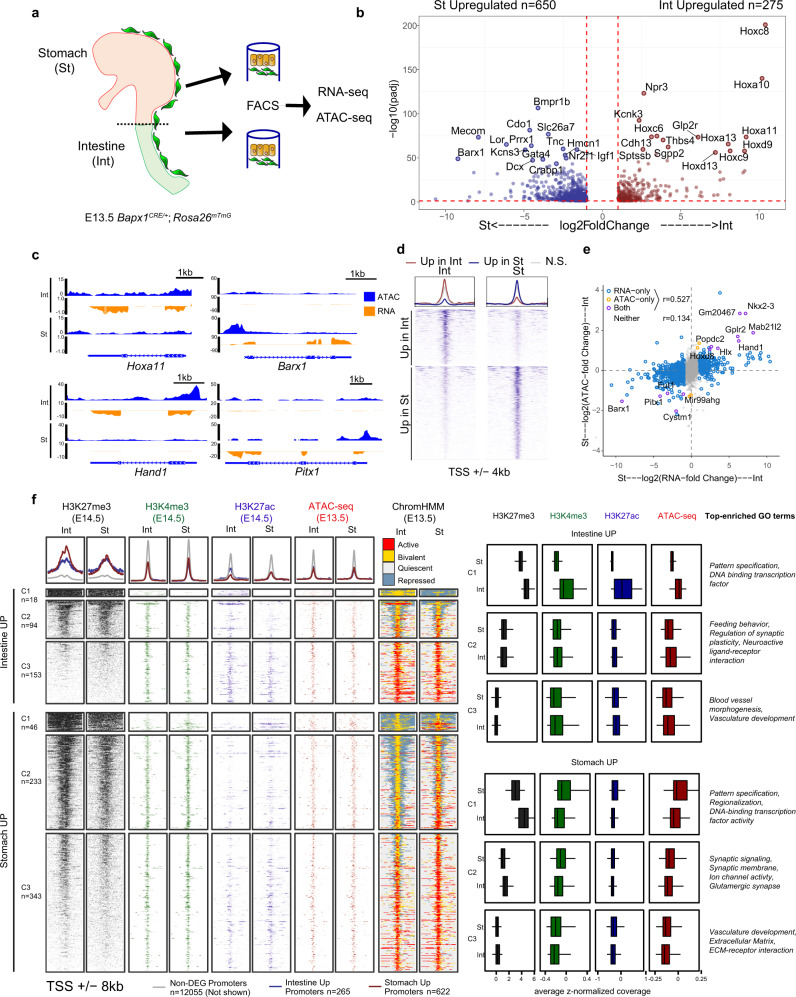
Epigenetic regulation of mesenchymal niches in GI development. **a** Diagram of GFP + mesenchymal cell isolation from E13.5 *Bapx1*^*Cre/+*^;*Rosa26*^*mTmG*^ mice. **b** Differentially expressed genes (DEGs) in RNA-seq data from E13.5 intestinal (Int, blue) versus stomach (St, red) mesenchyme (*n* = 2 biological replicates per tissue). Dashed red lines show thresholds for FDR adjusted *p*-value = 0.05 and log2(fold-change) = ±1. The top 15 genes per tissue, ranked by *p*-value, are labeled. **c** RNA-seq and ATAC-seq signals at example genes with tissue-specific expression and accessibility in the intestine (left) and stomach (right). Tracks depict merged signals from *n* = 2 biological replicates. Negative values indicate reverse-strand signal. Gene models from the WashU browser refGene track are shown. **d** E13.5 mesenchymal ATAC-seq signals for Int and St. Top: aggregate profiles for differentially accessible regions (DARs) in St (blue), Int (red), and non-significantly (N.S.) differential regions are shown for Int (left) and St (right). Bottom: Heatmap of ATAC signals for Int (top) and St (bottom) DARs. Viewing range shows peak center ±4 kb. **e** Comparison of DE and DA gene promoters in St versus Int. Pearson’s *R* is reported. **f** Aggregate profiles show signals from ENCODE E14.5 histone ChIP-seq and E13.5 ATAC signals from Int (left) and St (right) at expressed gene promoters. DEGs are categorized and color-coded as above. Simplified ENCODE ChromHMM chromatin state annotations are centered on promoters of DEGs, as described in panel. Top enriched GO terms are listed beside their corresponding cluster. Box and whisker plots show median (center), 1st and 3rd quartiles (limits of box). Whiskers extended to largest value within 1.5* the interquartile range (defined by box). (*n* = number of peak regions in each corresponding cluster (left of heatmap)).

To define organ-specific transcriptional programs of mesenchymal niches during GI development, we isolated mesenchymal cells of the stomach and intestine, using *Bapx1*^*Cre/+*^;*Rosa26*^*mTmG*^ mice at embryonic day (E)13.5, just prior to the establishment of region-specific morphological changes (Fig. 1a). Debris were excluded based on forward and side scatter properties, and dead cells were removed based on staining with the cell viability dye, Sytox Blue (Fig. S1). RNA-seq of these mesenchymal populations revealed hundreds of differentially expressed genes (DEGs) between the stomach and intestine: 275 genes display increased intestinal expression, while 650 genes show high gastric expression (Fig. 1b and Fig. S2a). This analysis not only validates the expression of *Barx1*, known to be restricted to the stomach mesenchyme, but also identifies poorly described organ-specific mesenchymal genes. For example, transcriptional regulators such as *Mecom* in the stomach and *Hand1* in the intestine may play important roles during GI organ specification (Fig. 1b, c).

To determine whether these organ-specific transcriptional programs are regulated at the chromatin level, we conducted ATAC-seq^25^ with E13.5 gastric and intestinal mesenchymal cells isolated from *Bapx1*^*Cre/+*^;*Rosa26*^*mTmG*^ mice, identifying 157,015 accessible chromatin regions in the stomach and intestine. Among these chromatin regions, 1635 (1.04%) were identified as differentially accessible regions (DARs), which include 1029 regions with increased accessibility in the stomach and 606 regions with increased accessibility in the intestine (Fig. 1d). These data suggest that during early development, the gastric and intestinal mesenchyme exist in a broadly permissive and highly similar chromatin state, sharing over 155,000 accessible regions between them (Fig. S2b, S2c). This remarkable similarity in chromatin accessibility is reminiscent of the broadly permissive chromatin pattern observed in the adult intestinal epithelium^26^, suggesting that mesenchymal cells at this stage may indeed possess an innate lineage plasticity.

### GI lineage-specific regulators show differences in chromatin accessibility

Despite the highly similar chromatin accessibility landscapes between the stomach and intestine, we observed a correlation between transcription and accessibility of promoters that are either differentially expressed or in proximity (within 1 kb) of a DAR (Pearson’s *r* = 0.527 for DEG or DAR-proximal promoters, versus *r* = 0.134 for gene promoters without significant changes in expression; *p* < 2.2e−16) (Fig. 1e). Further, DEGs were more likely to be associated with promoter proximal DARs than expected by chance (11.2-fold, hypergeometric *p* < 2.7e−20 for ±1 kb). Indeed, we found known mesenchymal regulators of gastric and intestinal fate, such as *Barx1*^7^ and *Nkx2–3*^27^, that display tissue-specific differences in both expression and promoter accessibility, suggesting that mesenchymal chromatin accessibility may regulate the expression of lineage-determining genes, despite broad similarities in chromatin accessibility (Fig. 1e).

### Chromatin bivalency may explain the broadly permissive chromatin architecture

Importantly, the majority of DEG promoters were associated with modest or non-significant changes in chromatin accessibility, with 50% of DEG promoters exhibiting <1.15-fold change in ATAC-seq signal, indicating that factors beyond accessibility may contribute to their regulation (Fig. 1b, e). We theorized that histone modifications could inform epigenetic mechanisms that regulate tissue-specific transcription independent of accessibility. Accordingly, we investigated histone modifications at the promoters of DEGs using ENCODE histone ChIP-seq and chromHMM chromatin state predictions from E14.5 whole mouse intestine and stomach^28,29^. We specifically focused on H3K27ac (associated with active regulatory elements), H3K27me3 (associated with Polycomb-mediated repression), and H3K4me3 (associated with active and poised promoters). Overall, DEG promoters were enriched for H3K27me3 compared to non-DEG promoters, and clustering of DEGs based on H3K27me3 revealed three distinct clusters for both sets of DEGs: C1—broad H3K27me3 peaks, C2—high focal H3K27me3, and C3—low focal H3K27me3. Interestingly, H3K4me3 is present at the TSS of all clusters in stomach and intestine DEGs. In C3, H3K4me3 coincides with H3K27ac, indicative of active promoters. In C1 and C2, H3K4me3 overlaps with H3K27me3 (Fig. 1f). The co-occupancy of H3K4me3 and H3K27me3 (seen at our DEG gene promoters in both tissues) is a hallmark of bivalent chromatin, wherein repressive and activating chromatin marks occupy the same locus, priming the downstream gene for transcriptional activation^30,31^.

Given the co-occupancy of H3K4me3 and H3K27me3 at DEG promoters, we hypothesized that mesenchymal chromatin bivalency may play a key role in GI regionalization and that resolution of bivalent chromatin may lead to activation of tissue-specific transcriptional programs. Indeed, annotating DEG clusters with ChromHMM maps validates that C1 and C2 promoters are bivalent^28,29^. We observed that a substantial fraction of stomach-upregulated gene promoters (33%, 233/622) switch from bivalent in the intestine to active in the stomach, but this pattern is absent for genes upregulated in the intestine (Fig. 1f). This data suggests that chromatin bivalency may be a key mechanism for regulating region-specific mesenchymal gene expression, implying a potential role for Polycomb-mediated restriction of stomach-associated genes in the intestinal mesenchyme.

### Loss of mesenchymal PRC2 alters gastrointestinal epithelial fate and function

**Fig. 2:**
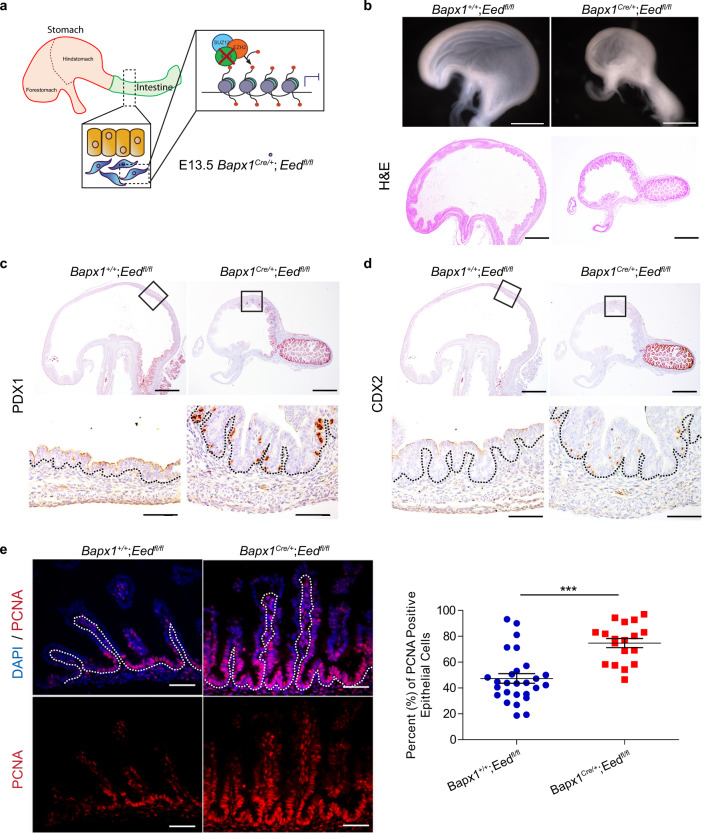
Mesenchymal loss of PRC2 disrupts GI epithelial fate and function. **a** Diagram of mesenchymal PRC2 ablation (*Bapx1*^*Cre/+*^;*Eed*^*fl/fl*^). **b** Whole mount imaging (top), and Haematoxylin and Eosin staining (bottom) of KO and control mice (*Bapx1*^*+/+*^;*Eed*^*fl/fl*^) at E17.5. **c**, **d** Immunohistochemistry against PDX1 (**c**) and CDX2 (**d**) shows expression of both markers in the epithelial layer of the proximal hindstomach (*n* = 3 biologically independent samples for each genotype). **e** PCNA immunofluorescence shows epithelial proliferation in E17.5 mice. Average percentage of proliferating intestinal epithelial cells per villus across *n* = 8 *Bapx1*^*+/+*^;*Eed*^*fl/fl*^ mice and *n* = 5 *Bapx1*^*Cre/+*^;*Eed*^*fl/fl*^ mice (duodenal tissues, ±SEM, ***P < 0.001 by Student’s *t*-test, two-tailed). Source data are provided as a source data file. Scale bars represent 50 µm. Dashed lines separate the epithelium (Epi) and mesenchymal cells (Mes).

Based on our finding of tissue-specific H3K27me3 at DEGs versus non-DEGs, and the observed chromatin bivalency at DEGs (Fig. 1f), we hypothesized that loss of H3K27me3 would impair the resolution of chromatin bivalency and alter accessibility at lineage-defining genes, leading to permissive chromatin, active transcription, and disrupted regionalization. To test the requirement of H3K27me3 in GI specification and regionalization, we genetically inhibited PRC2 function in the mesenchyme of the primitive gut tube by deleting Embryonic Ectoderm Development (*Eed*), a core component of PRC2 (*Bapx1*^*Cre/+*^;*Eed*^*fl/fl*^) (Fig. 2a). Immunofluorescence staining of H3K27me3 showed that mesenchymal PRC2 is disrupted upon *Eed* deletion (Fig. S3a, S3b).

Notably, the loss of PRC2 significantly reduced both stomach size and gut length, leading to misshapen structures on the luminal surface of the hindstomach (Fig. 2b and Fig. S4a). To determine if PRC2 disruptions result in altered regionalization, we investigated several regional markers of gastric and intestinal identity. Notably, stomach epithelial cells ectopically expressed CDX2^32,33^ (an intestinal marker) and PDX1^34^ (a distal hindstomach and proximal intestinal marker) (Fig. 2c, d). These changes are occurring in epithelial tissues, despite mesenchymal ablation of PRC2. This suggests that mesenchymal PRC2 may control intercellular communication to direct regional fate decisions. We also analyzed the intestine but observed no obvious changes in either cell death or intestinal identity (Fig. S4b–d). Instead, we found a significant increase in intestinal epithelial proliferation in response to mesenchymal PRC2 loss (Fig. 2e). Together, our data suggest that mesenchymal PRC2 may control GI epithelial fates and proliferation through intercellular communication.

### PRC2 controls the expression of key lineage-defining genes

**Fig. 3:**
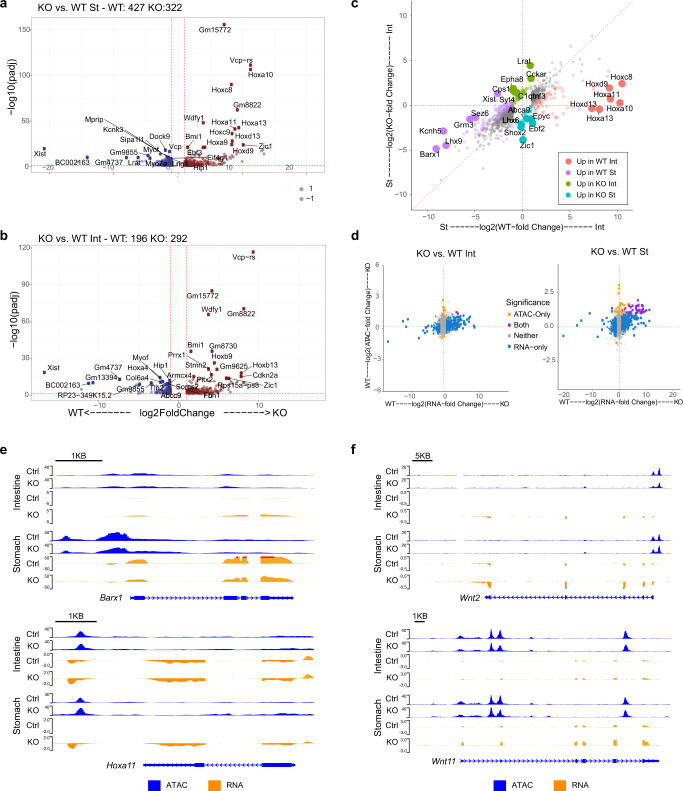
PRC2 loss alters gene expression with and without changes in chromatin accessibility. **a**, **b** DEGs between control and PRC2-Knockout (KO) Stomach (St) (**a**) and Intestine (Int) (**b**). **c** Comparison of Int and St DEGs in control (*x*-axis) and PRC2-knockout (*y*-axis) tissues. Only DEGs identified in control Int versus St and genes whose differential expression was significantly altered in PRC2-knockout Int versus St are shown and categorized by color as described in the panel. Top six genes per category are labeled. Dashed red line shows diagonal. **d** Correlation of promoter ATAC-seq signal and gene expression between PRC2-KO and control tissues. **e** Example tracks (*Barx1* and *Hoxa11*) showing lineage-specific genes which alter chromatin accessibility (blue) and transcription (orange). **f** Example tracks (*Wnt2* and *Wnt11*) demonstrating genes which alter transcription independent of chromatin accessibility upon PRC2 loss.

To understand the mechanisms by which PRC2 controls GI regionalization and intercellular communication, we analyzed chromatin accessibility and transcriptional profiles of gastric and intestinal mesenchymal cells in our *Eed* KO model by isolating mesenchymal cells from *Bapx1*^*Cre/+*^;*Eed*^*fl/fl*^;*Rosa26*^*mTmG*^ mice at E13.5. We first found that mesenchymal *Eed* expression is significantly reduced upon *Eed* deletion (Fig. S3c). Analyzing the transcriptional effects of PRC2 loss in both organs, we identified 922 DEGs (749 DEGs in the stomach, 488 DEGs in the intestine, with 254 common to both organs) (Fig. 3a, b). Genes sensitive to *Eed* deletion were significantly enriched for DEGs identified between control organs (232/922; *p* < 2.68e−77; hypergeometric test), and differences in organ-specific gene expression were diminished upon *Eed* KO (Fig. 3c and Fig. S5a). 19.6% of genes upregulated in the *Eed* KO stomach were enriched in the control intestine relative to the stomach, while 31.5% of genes upregulated in the *Eed* KO intestine were enriched in the control stomach (Supplementary Data 1, KO.vs.WT.Int and KO.vsWT.St.). These examples include organ-specific TFs such as *Hoxa11* and *Barx1* (Fig. 3e). Intestine-upregulated DEGs were twice as likely to be susceptible to PRC2 loss compared to stomach-upregulated DEGs (68/275 intestine DEGs versus 90/650 stomach DEGs, Fishers exact test *p* = 0.0001). These findings indicate that PRC2 regulates the expression of lineage-determining factors in an organ-specific manner, and mesenchymal PRC2 loss may fail to properly define organ fate prior to regionalization, leading to organs becoming more similar to one another.

### PRC2 controls gene expression dependent on/independent of chromatin accessibility

To understand whether these transcriptional changes and loss of regional identity are mediated by changes in chromatin accessibility, we performed ATAC-seq with mesenchymal cells isolated from *Bapx1*^*Cre/+*^;*Eed*^*fl/fl*^;*Rosa26*^*mTmG*^ mice. Differential analysis of ATAC-seq regions identified 2229 (1.4%) DARs, with 1983 DARs (720 up and 1263 down in KO versus WT) identified for stomach and 478 DARs (160 up and 328 down in KO versus WT) identified for intestine (Fig. S5b). Of the 2229 *Eed* KO-sensitive DARs, 134 (6.0%) overlapped with DARs identified between WT stomach and intestine (Supplementary Data 2). The small number of DARs identified following PRC2 loss was in agreement with a recent study in mouse embryonic stem cells, which demonstrated minimal changes in chromatin accessibility at Polycomb-occupied gene promoters following ablation of PRC1/PRC2^35^. Thus PRC2’s function, independent of widespread chromatin accessibility changes, further supports the idea that promoters of lineage-determining genes are maintained in an accessible state prior to regionalization. However, it is important to highlight that PRC2 loss does lead to a number of changes in chromatin accessibility.

To examine the enhancer and promoter context of accessibility changes, we annotated DARs using the ENCODE collection of candidate cis-regulatory elements (cCREs) in the mouse. Based on cCRE annotations, we found that accessibility changes in WT tissues are predominantly observed at distal enhancers, whereas accessibility changes following PRC2 loss occur more at promoters and promoter-proximal enhancers (Fig. S5c). Knowing that PRC2 disrupts regional identity and alters chromatin accessibility, we explored whether transcriptional changes are associated with changes in chromatin accessibility. Similar to our observation for control stomach versus intestine, DEGs between control versus mutant intestine or control versus mutant stomach were more strongly correlated with differential ATAC signals than non-significant genes. The majority of *Eed* KO DEGs were associated with modest changes in accessibility: 50% of DEG promoters exhibiting <1.13 and <1.16-fold change in ATAC-seq signal following *Eed* KO in stomach and intestine, respectively (*R* = 0.426 and *R* = 0.492 for DEG or DAR-proximal promoters, versus *R* = 0.154 and *R* = 0.173 for gene promoters without significant changes in expression in intestine and stomach, respectively; Fig. 3d; Supplementary Data 3). Interestingly, while only two genes (*Cdkn2b* and *Hoxb9*) exhibited both chromatin accessibility and expression changes in the intestine, 33 genes in the stomach showed concordant changes in promoter accessibility and gene expression. These genes include Hox family TFs, implying PRC2-mediated regulation of mesenchymal *Hox* genes in development (Supplementary Data 3). This data suggests that PRC2 may also regulate gene expression that is dependent on chromatin accessibility.

### Loss of PRC2 abnormally activates GI mesenchymal niche signals

To better understand how PRC2-mediates intercellular communication in the developing gut, we analyzed the differential expression of secreted ligands, specifically focusing on key developmental signaling pathways involved in controlling fate and proliferation in the GI system^2^. Indeed, we found their altered expression (Fig. S6). Wnt is a particularly interesting target, since activation of Wnt signaling is required to maintain intestinal fate, while its suppression is associated with gastric identity^7^. Further, mesenchymal Wnt ligands promote intestinal epithelial proliferation^15–24^. Indeed, examination of our RNA-seq data revealed significantly increased levels of *Wnt2* and *Wnt11* expression in intestine versus stomach (Supplementary Data 1) and we verified that this increase in *Wnt2* expression persists beyond regionalization by performing qRT-PCR for *Wnt2* with isolated mesenchymal cells at E16.5 (Fig. S7a). Of note, while *Wnt2* expression increased in PRC2-deleted cells, no significant differential changes in chromatin accessibility were observed, with both controls and knockouts possessing open chromatin at the promoter of *Wnt2*, suggesting that PRC2 controls expression of *Wnt2* independent of chromatin accessibility (Fig. 3f).

### Reduced mesenchymal Wnt secretion rescues fate and proliferation of GI epithelial cells

**Fig. 4:**
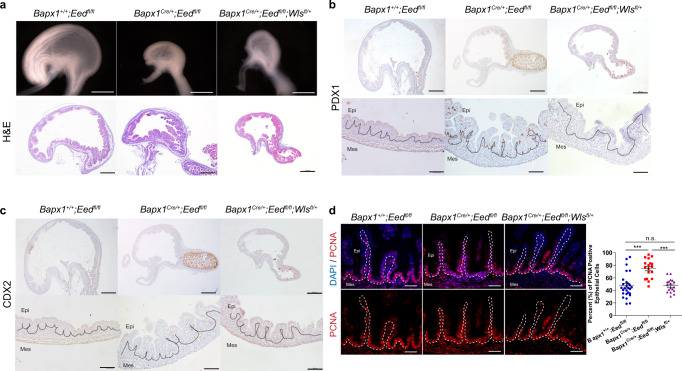
Reduced mesenchymal Wnt secretion restores gastric fate and intestinal proliferation in epithelial cells. **a**–**d** *Bapx1*^*+/+*^;*Eed*^*fl/fl*^ mice (left), *Bapx1*^*Cre/+*^;*Eed*^*fl/fl*^ mice (middle), and *Bapx1*^*+/+*^;*Eed*^*fl/fl*^;*Wls*^*fl/+*^ mice (right). **a** Whole mount imaging and Haematoxylin and Eosin staining demonstrating gastric morphology of each genotype (*n* = 3 biologically independent samples for each genotype). CDX2 (**b**) and PDX1 (**c**) immunohistochemistry showing regionalization pattern in hindstomachs of mice (*n* = 3 biologically independent samples for each genotype). **d** PCNA immunofluorescence shows proliferation patters for each genotype. Epithelial PCNA data was quantified by determining the percent of PCNA positive epithelial cells per villus where each dot represents an individual villus (duodenal tissues, *n* = 8 *Bapx1*^*+/+*^;*Eed*^*fl/fl*^ mice, *n* = 5 *Bapx1*^*Cre/+*^;*Eed*^*fl/fl*^ mice and *n* = 4 *Bapx1*^*Cre/+*^;*Eed*^*fl/fl*^;*Wls*^*fl/+*^ mice, ***P < 0.001, n.s—not significant by Student’s *t*-test, two-tailed). Source data are provided as a source data file. *Bapx1*^*+/+*^;*Eed*^*fl/fl*^ and *Bapx1*^*Cre/+*^;*Eed*^*fl/fl*^ data were described in Fig. 2, with data from *Bapx1*^*+/+*^;*Eed*^*fl/fl*^;*Wls*^*fl/+*^ mice appended to these counts. Scale bars represent 50 µm. Dashed lines separate the epithelium (Epi) and mesenchymal cells (Mes).

To determine if alterations in mesenchymal Wnt ligand production cause the observed epithelial phenotypes (Fig. 2), we conditionally deleted a single copy of *Wntless* (*Wls*)^36^ in mesenchymal cells of a PRC2-deficient background (*Bapx1*^*Cre/+*^;*Eed*^*fl/fl*^;*Wls*^*fl/+*^). Notably, the loss of a single copy of *Wls* rescued the gastric regionalization and intestinal proliferation defects observed in *Bapx1*^*Cre/+*^;*Eed*^*fl/fl*^ mice (Fig. 4a–d). Interestingly, the heterozygous deletion of *Wls* was unable to rescue gastric morphology, suggesting that mesenchymal PRC2 likely controls GI morphogenesis independently of Wnt signaling. Nevertheless, this data demonstrates that PRC2 controls mesenchymal Wnt niche signals to regulate intercellular communication in the developing GI tract, ultimately ensuring proper regionalization and function of neighboring epithelial cells. Collectively, these findings reinforce our assertion that epigenetic states influence intercellular communication in both organogenesis and adult stem cell homeostasis.

### Mesenchymal PRC2 mediates organ specification through maintenance of bivalent chromatin

**Fig. 5:**
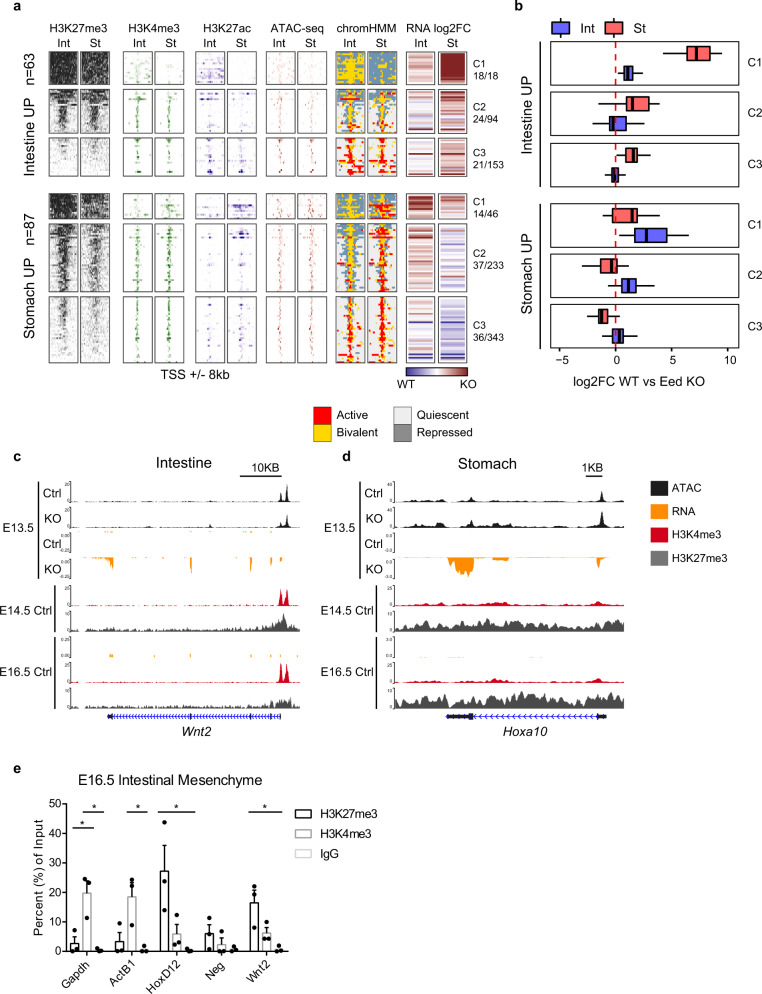
Promoter bivalency regulates intercellular communication in GI development. **a** Aggregate profiles and chromatin state plots, as in Fig. 1f, for genes whose differential expression between Int and St is altered upon PRC2-knockout (as described in Fig. 2a, b). Gene promoters were classified based on their DEG status between control Int and St. Simplified chromatin state maps are shown for E14.5 for both St and Int. **b** Log2 Fold Change of control stomach-enriched (red) and intestine-enriched (blue) genes upon PRC2 loss in each organ, clustered by degree of H3K27me3 at the promoter (as in Fig. 1f). Box and whisker plots show median (center), 1st and 3rd quartiles (limits of box). Whiskers extended to largest value within 1.5* the interquartile range (defined by box). **c**, **d** Tracks overlaying transcription and chromatin accessibility of *Wnt2* in the intestinal mesenchyme (**c**) and *Hoxa10* in the stomach mesenchyme (**d**) in E13.5 control and PRC2-knockout mice. ENCODE ChIP-seq data for H3K27me3 and H3K4me3 (E14.5 and E16.5) and RNA-seq (E16.5) are shown for the respective tissues. **e** ChIP-qPCR of H3K27me3 and H3K4me3 in mesenchymal cells enriched from E16.5 mice. Multiplicity adjusted *p*-values were calculated by two-way ANOVA with Bonferroni post-tests (*n* = 3 biologically distinct sets of embryos, 8 embryos per litter were pooled for each *n*, ±SEM, *P < 0.05). Source data are provided as a source data file.

To define PRC2-mediated epigenetic regulation of gut mesenchymal niche signals such as Wnt ligands, we examined H3K27me3, H3K4me3, and H3K27ac ChIP profiles at the promoters of organ-specific genes that are sensitive to PRC2 loss in control E14.5 stomach and intestine^28,29^. Organ-specific DEGs susceptible to PRC2 loss were predominantly those with high levels of promoter H3K27me3 (Fig. 5a). H3K27me3-high clusters (C1 and C2) were 3.8-fold and 1.9-fold enriched for PRC2 sensitive genes than H3K27me3-low (C3) clusters in the intestine and stomach, respectively (Fishers exact test *p* = 9.5e−6 and *p* = 0.007, respectively). Intestine-upregulated DEGs demonstrated significantly larger fold-change increases in the mutant stomach than the mutant intestine; stomach-upregulated DEGs showed greater fold-change increases in the mutant intestine (Fig. 5b). Together, these indicate a role for PRC2 in mediating repression of lineage-inappropriate genes in an organ-specific manner. We also observed an enrichment of both H3K27me3 and H3K4me3 at the promoters of PRC2-sensitive DEGs, suggesting that PRC2-sensitive genes are bivalent chromatin domains (Fig. 5a). To further investigate this possibility, we stratified PRC2-sensitive DEGs in each tissue based on bivalency status and the change in promoter chromatin accessibility. We found that approximately 54% (250/464) of PRC2-sensitive genes in the intestinal mesenchyme are marked by bivalent chromatin modifications, and in the stomach, approximately 37% of PRC2-sensitive genes are marked by bivalent chromatin modifications (Fig. S8a). To better understand PRC2-mediated bivalency, we performed a GO enrichment analysis in the stomach and intestinal PRC2-sensititive bivalent genes. We found that DNA-binding transcription factor activity, and embryonic developmental process, such as regionalization, patterning and morphogenesis, are highly enriched in the PRC2-sentitive bivalent genes (Fig. S8b). Interestingly, in the intestinal mesenchyme, Wnt was also enriched among the PRC2-sensitive bivalent genes (term name: GO:0016055 Wnt Signaling Pathway, log2 fold enrichment = 2.18, *P* = 0.00057). For example, while PRC2-sentive genes without promoter bivalency such as *Hoxa10* increased chromatin accessibility upon PRC2 loss, promoter bivalency and open chromatin is present at many other PRC2-sentitive genes, including *Wnt2* (Fig. 5c, d and Fig. S7b). We also validated that that promoter bivalency of *Wnt2* exists specifically in gut mesenchymal cells through ChIP-qPCR (Fig. 5e). Together, these data demonstrate that PRC2 likely controls intercellular communication in the developing gut, largely through maintenance of accessible bivalent chromatin domains, to establish proper regionalization and function of neighboring epithelial cells. Importantly, these findings suggest that PRC2 can maintain bivalent chromatin states to control transcription without altering chromatin accessibility. This likely explains why gene expression is restricted to tissue-specific patterns, yet chromatin accessibility is broadly similar between GI organs.

### PRC2 ablation in pericryptal mesenchymal cells disrupts stem cell homeostasis

**Fig. 6:**
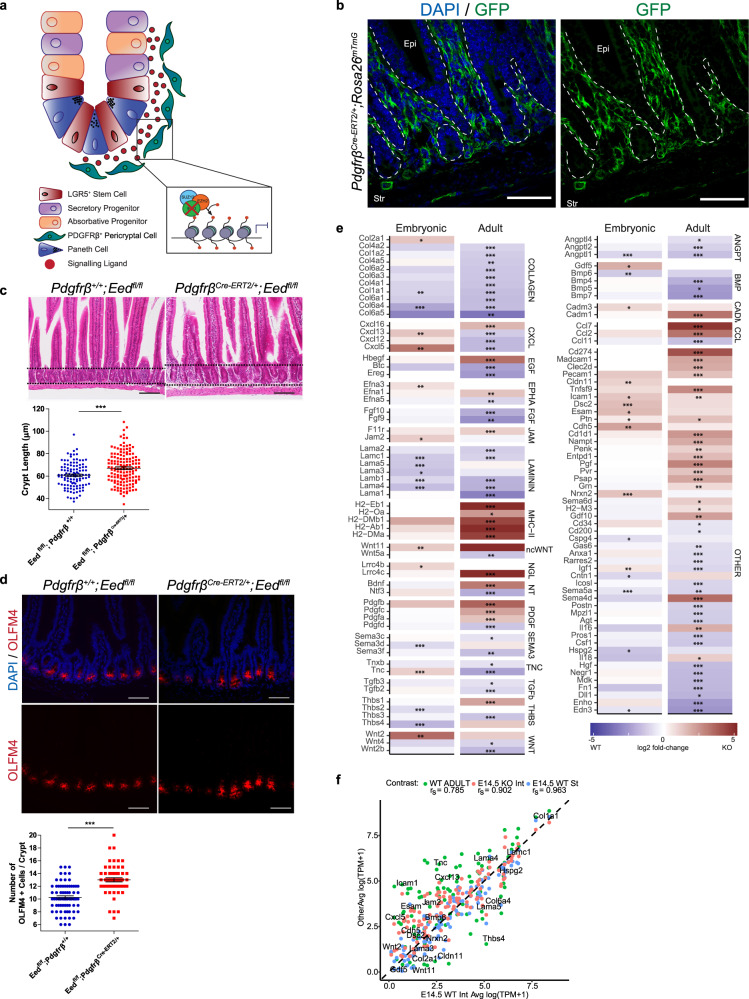
Pericryptal loss of PRC2 alters stem cell homeostasis. **a** Schematic illustrating the loss of PRC2 in pericryptal cells comprising the intestinal stem cell niche. **b** GFP expression in *Pdgfrβ*^*Cre-ERT2/+*^;*Rosa26*^*mTmG*^ mice upon induction with tamoxifen (100 mg/kg, 20 mg/mL). **c**–**e** *Pdgfrβ*^*+/+*^;*Eed*^*fl/fl*^ (left) and *Pdgfrβ*^*Cre-ERT2/+*^;*Eed*^*fl/fl*^ (right) (*n* = 3 biologically independent samples for each genotype). **c** Haematoxylin and Eosin staining of duodenal sections. Black dotted lines outline the crypts of each mouse. Crypt length (µm) was quantified by measuring the longest edge of each crypt using Fiji. Each point represents an individual crypt measurement (*n* = 4 *Pdgfrβ*^*+/+*^;*Eed*^*fl/fl*^ mice and *n* = 5 *Pdgfrβ*^*Cre-ERT2/+*^;*Eed*^*fl/fl*^ mice, ±SEM, ***P < 0.001 by Student’s *t*-test, two-tailed). Source data are provided as a source data file. **d** OLFM4 immunofluorescence identifying stem cells in the crypt base. Number of OLFM4-positive cells were quantified per crypt, with each dot representing a single measurement (duodenal tissues, *n* = 3 biologically independent samples for each genotype, ±SEM, ***P < 0.001 by Student’s *t*-test, two-tailed). Source data are provided as a source data file. White dashed lines separate the epithelium (Epi) and stromal cells (Str). All scale bars represent 50 µm. **e** Differential expression of all secreted ligands following *Eed* KO in adult and embryonic intestinal mesenchymal cells. * denotes significantly DE in between WT and *Eed* KO at the respective age. *p < 0.05; **p < 0.01; ***p < 0.001. **f** Correlation of expression of secreted ligands (log(TPM + 1)) comparing E14.5 WT Int (*x*-axis) versus WT Adult Int, E14.5 KO Int, and E14.5 WT St (*y*-axis; green, red, and blue dots, respectively). Ligands shown are as in **e**. Genes identified as PRC2-sensitive in E14.5 WT Int are labeled. TPM values were calculated from spike-in normalized counts and averaged between replicates. Spearman’s rho is reported for each axis.

During adult homeostasis, increasing evidence suggests that mesenchymal cells in proximity to epithelial stem cells constitute a key stem cell niche that secretes niche factors such as Wnt ligands^17,19–22^. As we demonstrated PRC2-mediated regulation of Wnt ligand expression in mesenchymal cells during development (Figs. 3–5 and Fig. S7), we hypothesized that PRC2 in pericryptal mesenchymal cells may also regulate stem cell niche signals. Recently, we identified GI mesenchymal cell populations that co-express *Pdgfrβ* and *Foxl1*, both known telocyte markers^21,22,37^. We first validated the expression *Pdgfrβ* in pericryptal cells adjacent to intestinal crypts (Fig. 6a, b). To determine whether PRC2 is involved in regulation of the adult ISC niche, we conditionally deleted *Eed* in *Pdgfrβ*-expressing cells (*Pdgfrβ*^*Cre-ERT2/+*^;*Eed*^*fl/fl*^) (Fig. 6a, b and Fig. S9a). After 5 days of tamoxifen treatment and 2 days of rest, *Pdgfrβ*^*Cre-ERT2/+*^;*Eed*^*fl/fl*^ and *Pdgfrβ*^*+/+*^;*Eed*^*fl/fl*^ mice were sacrificed and analyzed. *Pdgfrβ*^*Cre-ERT2/+*^; *Eed*^*fl/fl +*^ mice demonstrated a significant increase in crypt length (*p* < 0.001) relative to CRE-negative controls, as well as an increase in the number of stem cells marked by OLFM4 (*p* < 0.01) (Fig. 6c, d). Interestingly, no change was observed in the number of proliferative cells, as marked by PCNA, suggesting that loss of PRC2 in pericryptal cells specifically increases the pool of ISCs (Fig. S9b). To address whether PRC2 regulation of Wnt ligand expression is specific to the developing intestine or common to both development and stem cell homeostasis, we generated a mouse model to restrict Wnt ligand secretion in pericryptal cells of the adult intestine in PRC2 loss (*Pdgfrβ*^*Cre-ERT2/+*^;*Eed*^*fl/fl*^;*Wls*^*fl/+*^). Interestingly, we observed no rescue of crypt length in this model, suggesting that mesenchymal PRC2 may control adult stem cell niche signals through a mechanism different from that in gut development (Fig. S9c).

To define further PRC2-mediated regulation of adult stem cell niche signals, we isolated pericryptal cells from *Pdgfrβ*^*Cre-ERT2/+*^;*Eed*^*fl/fl*^;*R26*^*mTmG*^ mice and performed RNA-seq. We queried DEGs against a curated database of secreted ligands^38^ and observed distinct effects on the expression of many morphogens, including WNT and BMP ligands. For example, PRC2 loss in the embryonic mesenchyme resulted in the upregulation of *Wnt2* and *Wnt11*, whereas its loss in the adult mesenchyme led to the downregulation of *Wnt4*, *Wnt5a*, and *Wnt2b*, and the upregulation of *Bmp4*, *Bmp5*, and *Bmp7* (Fig. 6e). Overall, we found the expression pattern (normalized transcript per million (TPM)) of PRC2-sensitive secreted ligands to be dissimilar between the adult and E14.5 WT intestinal mesenchymal cells (Spearman’s Rho = 0.785) relative to other E14.5 samples (Rho = 0.902 for WT E14.5 versus KO E14.5 int; Rho = 0.963 for WT E14.5 Int versus WT E14.5 St) (Fig. 6f). Our data shows that PRC2 loss in adult pericryptal cells results in the dysregulation of a largely distinct repertoire of secreted ligands, likely due to the dynamic regulation of secreted ligand expression between adult and embryonic developmental timepoints (Fig. 6e, f, and Figs. S6, S10). Taken together, our work demonstrates that PRC2 controls mesenchymal niche signals involved in gut specification, progenitor proliferation, and morphogenesis, as well as stem cell homeostasis.

## Discussion

Our chromatin and transcriptional profiling of pre-regionalization mesenchymal tissues sheds light on the epigenetic mechanisms of tissue crosstalk driving regionalization in the gastrointestinal system. A small number of key lineage-defining genes, which include key transcription factors such as *Hox* genes and *Barx1*, possess tissue-specific patterns of accessibility, suggesting an important relationship between chromatin accessibility and control of tissue-specific gene expression. However, we were surprised to discover that, prior to regionalization, the chromatin of the stomach and intestinal mesenchyme has highly similar patterns of chromatin accessibility. This striking similarity is interesting, as the broad accessibility may underlie an innate lineage plasticity in the developing gut tube. Given the low number of DE genes that simultaneously show unique patterns of chromatin accessibility, we reasoned that another factor could be involved in regulating gene expression independently of accessibility. Indeed, we show here that many DE genes are marked by bivalent chromatin, which canonically restricts gene expression while maintaining an open chromatin state. We found that impairing H3K27me3 in mesenchymal cells through inhibition of PRC2 had mild effects on chromatin accessibility, but significantly changed gene expression patterns. In accordance with these changes, we found that PRC2 ablation impairs both regionalization and stem cell functionality.

Of note, PRC2 in the intestinal epithelium is known to play key roles in stem cell proliferation and differentiation^39–41^. Mechanistically, developmental genes silenced by H3K27me3 become reactivated upon PRC2 loss. Interestingly, the H3K4me3 levels at bivalent promoters of these genes influence the extent of their reactivation, demonstrating the complex and precise PRC2-mediated regulation of gut epithelial transcriptional programs^40,42^. Accumulating evidence suggests that gut mesenchymal cells secrete niche ligands to regulate both organ development and stem cell homeostasis, demonstrating the importance of tissue crosstalk^43,44^. Since these mesenchymal niche signals must be dynamically regulated, epigenetic regulation would likely play a key role. However, the role of epigenetic regulators in mesenchymal niches have been poorly understood. By specifically targeting mesenchymal PRC2, our work demonstrates that PRC2 controls the gene expression of key niche ligands and transcription factors involved in gut development and stem cell homeostasis.

Indeed, mesenchymal PRC2 loss induced altered epithelial fates and function, indicative of changes in intercellular communication, which we have attributed these changes to increases in Wnt ligand expression during development. Wnt signaling must be downregulated by mesenchymal factors such as Barx1 in the stomach for its proper identity^7^. Indeed, mesenchymal PRC2 loss led to the abnormal activation of intestinal marker genes, such as *Cdx2* in the stomach. Although the proliferation of intestinal progenitors is significantly increased upon PRC2 loss, we observed no obvious changes in intestinal identity. While Wnt ligands are already active in the developing intestine, their abnormal activation upon PRC2 loss may not influence intestinal identity.

To define the role of mesenchymal PRC2 during adult intestinal stem cell homeostasis, we also conditionally deleted *Eed* in the adult intestinal mesenchyme. While we observed an increased number of intestinal stem cells upon PRC2 loss as expected, the expression of more diverse sets of secreted ligands was altered in the adult compared to its defined regulation in development. Additional studies would be required to further define PRC2-mediated regulation of mesenchymal stem cell niche signals. A recent study has shown that the intestinal mesenchymal niche also controls tumor initiation^45^, and it would therefore be interesting to examine whether chromatin patterns of tumor-associated mesenchymal niches change during tumorigenesis and whether these changes influence tumor initiation and/or progression.

Collectively, our data suggest two key findings. First, PRC2 maintains bivalent chromatin domains in the developing gut, and second, PRC2 specifically regulates intercellular communication to ensure proper regionalization and function of neighboring epithelial cells. These observations stand as significant, clear evidence that epigenetic patterns do not simply regulate intrinsic cell properties but control the expression of crucial niche factors that support neighboring tissues. Disruptions in cellular function may simply be caused by the specific placement of histone modifications in neighboring cells, offering an additional layer of regulation that should be considered when examining intercellular communication patterns.

## Methods

### Mouse lines

All mice were handled in accordance with the rules and regulations of the Canadian Council on Animal Care Guidelines for Use of Animals in Research and Laboratory Animal Care under protocols approved by the Animal Care Committee at The Center for Phenogenomics (protocol: 19-0276H). *Pdgfrβ*^*Cre-ERT2/+*^, and *Eed*^*fl/fl*^ and *Bapx1*^*Cre/+*^ mice were received from the Sung lab at SickKids and Shivdasani lab at the Dana Farber Cancer Institute at Harvard Medical School, respectively. *Wls*^*fl/fl*^ and *Rosa26*^*mTmG*^ mice were purchased from Jackson Labs.

### Cell isolation for sequencing

Isolation of *Bapx1*-expressing mesenchymal cells from E13.5 embryos was achieved through sorting of GFP^+^ cells in *Bapx1*^*Cre/+*^;*Rosa26*^*mTmG*^ and *Bapx1*^*Cre/+*^;*Eed*^*fl/fl*^;*Rosa26*^*mTmG*^ mice via fluorescent activated cell sorting (FACS). GFP^+^ embryos were identified through fluorescence microscopy. Stomachs and intestines of each animal were separated through microdissection and placed in 2% fetal bovine serum (FBS, Sigma Aldrich, F1051) in ultra-pure PBS. Samples were then centrifuged at 4 °C for 5 min at 400*g*. Samples were digested in 3 ml of a 2:1 ratio of TrypLE Express (Gibco, 12604021) to ultrapure PBS until tissues clumped together (approximately 10 min). Tissue were then subjected to manual dissociation with a p200 pipette until a single-cell solution was observed. Samples were then neutralized with an equal volume of 2% FBS in ultrapure PBS and centrifuged at the conditions described above. Samples were washed in ultrapure 2% FBS and centrifuged again. After decanting the solution, cells were resuspended in ultrapure 2% FBS containing a 1:5000 dilution of Sytox Blue (Invitrogen, S34857), and filtered through 35 µm filter mesh (Fisher Scientific, 352235) into a polypropylene tube (Fisher Scientific, 352063) in preparation for FACS using the Sony SH800 BRV instrument. Cytobank 7.2 was used to analyze flow cytometry data.

Isolation of *Pdgfrβ*-expressing, GFP positive stromal cells was achieved by FAC sorting of *Pdgfrβ*^*Cre-ERT2/+*^;*Eed*^*fl/fl*^;*Rosa26*^*mTmG*^ and *Pdgfrβ*^*Cre-ERT2/+*^;*Rosa26*^*mTmG*^ mice (~10 weeks of age). Briefly, small intestines were isolated, opened, and washed in GB1 (HBSS (Gibco, 14175103) with 10% fetal calf serum (Gibco, 26010074) and 10 mM HEPES (Gibco, 15630080)). Villi were carefully scraped off using a glass slide and discarded. Remaining tissue was cut into small pieces, vortexed, and washed twice in GB1. Tissues were transferred to GB2 (GB1 with 10 mM EDTA) and shaken for 20 min (220 RPM) at 37 °C before being vortexed and washed through a 100 µm filter mesh to remove epithelial cells. This process was repeated twice and remaining tissues were washed in GB1 before being placed in digestion solution (20 ml RPMI (Gibco, 11875093), 1% Penicillin–Streptomycin (Gibco, 15140122), 10% FBS, 15 mM HEPES, 2 mg DNAse I (Worthington Biochemical, LS002139), 3.12 mg Protease IV, 58 mg Dispase II (Roche Diagnostics, 04942078001) for 1 h at 37 °C, vortexing every 15 min. The cell suspension was poured through a 70 µm strainer and centrifuged at 400 g for 5 min at 4 °C. The supernatant was removed and this wash was repeated. Cells were resuspended in 2% FBS with 1:5000 Sytox Blue and filtered through a 35 µm mesh in preparation for FACS using the Sony SH800 BRV instrument.

### RNA isolation and sequencing analysis

Total RNA isolation was completed using the RNeasy Micro Kit (Qiagen, 74004) following the manufacturer’s instructions. Prior to library preparation, spike-in RNA was added to control for variation in sample preparation (Lexogen, SIRV Set 3, 051). For embryonic samples, stranded Poly(A) mRNA libraries were made using the NEBNext Ultra II Directional RNA Library Prep Kit for Illumina (New England Biolabs, E7760) and underwent 100 bp paired-end sequencing using the HiSeq 2500. Adult RNA-seq libraries (unstranded) were prepared using the NEBNext Single-cell/low input RNA library prep kit (New England Biolabs, E6420). Libraries were sequenced to a median depth of 45 m read pairs per sample. Illumina base calling was performed using bcl2fastq v2.20. Raw reads were obtained in fastq format and trimmed for quality using Trimmomatic v0.32 before alignment with STAR v2.5.1b to mouse genome version GRCm38/mm10. Transcriptome annotations were built using GENCODE vM4. Both the genome and transcript annotations were modified to include SIRV spike-in sequences and transcript models. Normalized signal tracks were generated using STAR and visualized on the WashU epigenome browser.

Read counting was performed using featureCounts v1.5.0 (embryonic data) and v1.5.3 (adult data). Raw counts were spike-in batch normalized using RUV-seq v1.18.0. Differential analysis was performed using DESeq2 v1.24.0 in R. Cutoffs of pAdj < 0.05 (using pAdjustMethod = fdr) and log2FoldChange > 1 were used to define DEGs. To identify genes whose differential expression between Int and St were altered in PRC2-knockout, we used the contrast (Int.WT–St.WT)—(Int.KO–St.KO) with the cutoffs pAdj < 0.2 and log2FoldChange > 1.

### ATAC-seq and analysis

GFP^+^ cells were isolated from E13.5 *Bapx1*^*Cre/+*^;*Rosa26*^*mTmG*^ and *Bapx1*^*Cre/+*^;*Eed*^*fl/fl*^*;Rosa26*^*mTmG*^ via FACS in aliquots of 50,000 cells. ATAC-seq was performed using the Omni-ATAC-seq protocol^46^. Paired end reads were obtained in fastq format and trimmed using Trimmomatic v0.32 prior to alignment to GRCm38/mm10 using BWA-mem with default settings. Initial alignments were assessed for quality and processed using the ataqv pipeline v0.9.1 to retain high-quality autosomal aligned (hqaa) reads. Hqaa reads from replicate samples were pooled prior to peak calling with MACS2 v2.1.2 using the cutoffs -q 0.01–fe-cutoff 2.0–max-gap 250. A consensus peakset was obtained by merging peaks called from all tissues and conditions for downstream analysis. Read pairs were counted to the consensus peak-set using featureCounts v1.5.3. Differential analysis was performed using DESeq2 v1.24.0 in R. Cutoffs of pAdj < 0.05 (using pAdjustMethod = fdr) and abs(log2FoldChange) > 1 were used to define DARs.

### Combined ATAC and RNA-seq and chromatin state analyses

Preprocessed signal (bw format) and peaks (narrowPeak) format were downloaded from the ENCODE dataportal for H3K27ac, H3K27me3 and H3K4me3 ChIP-seq datasets for mouse Int and St at E14.5, E15.5, E16.5, and P0. Precalculated ChromHMM chromatin state annotations were obtained for E14.5 Int and St.

A set of putative St/Int promoters was obtained by merging ENCODE published mouse stomach and intestine H3K4me3 peaks from E14.5, E15.5, E16.5, and P0 (Supplementary Table 1). Gene promoters were approximated as the midpoint of the nearest H3K4me3 peak within 50 kb of the annotated TSS. Promoter-associated ATAC peaks were defined as the nearest consensus ATAC-seq peak within 50 kb of the approximated promoter (defined above). Per-region coverage of ChIP-seq/ATAC/ChromHMM annotations were obtained in tabular format from bigWig (or bed-converted bigWig for ChromHMM) using the convertMatrix function from deepTools v3.1.3 prior to clustering and visualization with R. Midpoints of ATAC-seq peaks and approximated promter H3K4me3 peaks were used for ATAC-seq peaks and genes, respectively.

### Quantitative reverse transcription PCR

E16.5 embryos were harvested, washed and dissected in ice-cold 1× PBS. Mutants were identified through phenotyping stomach shape and size. For each mutant, the stomach and intestine were mechanically separated. The forestomach was subsequently separated and removed. The hindstomach and intestinal segments were separately washed in 5 ml of 1× PBS with 150 µl of 0.5 M EDTA for 30 min at 4 °C while rotating. Samples were shaken monitored for separation of epithelial and mesenchymal layers every 5 min. Supernatant (containing epithelial tissue) was removed. Samples were washed and centrifuged at 100*g* (4 °C for 5 min). This process was then repeated. Once epithelial cells were removed, mesenchymal tissue was washed in PBS, the supernatant was removed, and Trizol (Thermo Fisher Scientific, 10296010) was added to lyse samples. Samples were homogenized for 2 min using a bead-beater, and RNA isolation was performed through phenol–chloroform extraction. cDNA Libraries were prepared using the Superscript III First-Strand Synthesis kit (Thermo Fisher Scientific, 18080093), and qRT-PCR was carried out using Power Sybr Green PCR Master Mix (Thermo Fisher Scientific, 4368577). Primer sequences are provided in Supplementary Table 2.

### Histology

For Hematoxylin and Eosin staining, paraffin-embedded tissues were sectioned at 5 microns (embryonic) and 8 microns (adult). Slides were deparaffinized with xylenes (Caledon Laboratory Chemicals, 9800-1-40) and rehydrated in ethanol before a 5 min treatment in Harris’s Hematoxylin (Electron Microscopy Sciences, 26041-06). After a brief (20 s) immersion into acid ethanol (0.3% HCl in 70% ethanol), slides were washed in water and place in 70% ethanol for 1 min. Slides were then treated with Eosin Y (Electron Microscopy Sciences, 26051-11) for 1 min, rinsed with water, dehydrated, and mounted.

### Immunohistochemistry

After isolation, samples were washed with ice-cold PBS and placed into 4% PFA to fix overnight at room temperature. Fixed samples were washed in PBS, and dehydrated in 70% ethanol for paraffin embedding. Embedded tissues were sectioned as described above. Slides were deparaffinized and rehydrated, and subject to antigen retrieval in sodium citrate buffer with 0.05% Tween-20 (Sigma, P9416) for 20 min in a steamer. Following antigen retrieval, slides were immersed in 3% hydrogen peroxide in methanol for 30 min, followed by a 5 min wash in 1× PBS. Sections were first blocked in 10% goat serum (Gibco, 16210072) for 1 h at room temperature, washed in PBS, then blocked in Avidin D (Thermo Fisher Scientific, 004303) for 15 min. Samples were washed in PBS, and blocked with Biotin (Thermo Fisher Scientific, 004303) for 15 min before being washed again. Samples were then incubated with primary antibodies at 4 °C overnight in a humidified chamber as follows: Mouse PDX1 (1:300; Developmental Studies Hybridoma Bank, F109-D12), mouse CDX2 (1:300; Biogenex, MU392A-UC). Slides were washed with PBS for 30 min, and incubated with biotin-conjugated secondary anti-mouse antibodies (1:200; Vector Laboratories, BA-9200) at room temperature for 1 h. Slides were washed for 30 min, and treated with ABC solution (Vector Laboratories, PK-6100) to detect the biotinylated antibody. After washing, slides were developed using 3,3-diaminobenzidine tetrahydrochloride (DAB) (Vector Laboratories, SK-4100). Slides were then counterstained with hematoxylin, dehydrated, and mounted.

### Immunofluorescence

Tissues, sectioning, and dehydration were completed in the same manner as Immunohistochemistry samples. Antigen retrieval was also performed as described above, with the exception of staining for OLFM4 and GFP, in which slides underwent antigen retrieval in 10 mM Tris-EDTA pH 9.0 (10 mM Tris, 10 mM EDTA). Slides were then washed in 1× PBS with 0.05% Tween-20, and blocked in 10% goat serum in this washing solution for 1 h at room temperature. Slides were washed in washing solution, and incubated in primary antibodies diluted in blocking solution at 4 °C overnight in a humidified chamber. Primary antibodies were used as follows: rabbit H3K27me3 (1:750; EMD Millipore, 07-449), mouse PCNA (1:300; Santa Cruz Biotechnology, sc-56), rabbit OLFM4 (1:100; Cell Signaling Technology, D6Y5A), mouse GFP (1:300; Santa Cruz Biotechnology, 9996). Goat anti-mouse Alexa 488 (Thermo Fisher Scientific, A11029), goat anti-mouse Alexa 568 (Thermo Fisher Scientific, A11031), and goat anti-rabbit Alexa 568 (Thermo Fisher Scientific, A11036) were used as secondary antibodies at 1:500 in washing buffer with nuclear Hoescht 33342 stain (1:1000; Thermo Fisher Scientific, 62249) for 1 h at room temperature. Slides were mounted with Fluoroshield Mounting Medium (Abcam, 104135) and sealed with clear nail polish for preservation.

### Tamoxifen treatment

Tamoxifen (Sigma-Aldrich, T5648) was dissolved in sunflower seed oil (Sigma-Aldrich, S5007-250ml) at a concentration of 20 mg/ml. Mice were treated with tamoxifen at a concentration of 100 mg/kg per day for five consecutive days via intraperitoneal injection. For *Pdgfrβ*^*Cre-ERT2/+*^; *Eed*^*fl/fl*^; and control (*Eed*^*fl/fl*^;*Pdgfrβ*^*+/+*^) analyses, mice (~10 weeks of age) were dissected two days after the final tamoxifen treatment.

### Fluorescent in-situ hybridization (FISH)

Single molecule FISH was performed using the RNAScope system from Advance Cell Diagnostics (ACD). The mouse-specific *Axin2* probe (400338-C2, ACD), as well as the positive and negative control probes, were designed by ACD. Freshly sectioned paraffin-embedded samples were stained following the manufacturer’s instructions. Images were acquired using the 20× (air) and 40× (water) objectives on the NikonA1R system. *Axin2* transcripts were quantified in epithelial cells using the “Find Maxima” function in Fiji with the detection threshold set to 700.

### ChIP-qPCR

E16.5 embryos were dissected as described above. For control litters, 8 embryos were collected per *n*. In cold 1× PBS, the intestines were opened by inserting fine forceps or needles into the lumen before being cut open from the outside. Opened intestines were placed in a tube of 2% FBS in PBS until all dissections were complete. Likewise, hindstomachs were separated from forestomachs and placed into 2% FBS in PBS. Washed tissues were immersed in 10 mM EDTA and rotated at 4 °C for 20 min. Tissues were washed and were suspended in a sucrose (54.9 mM) and D-sorbitol (43.4 mM) solution in 1× dPBS. Aamples were shaken by hand (2–3 cycles per second) for 5 min and filtered 70 µm strainer to remove epithelial cells. Mesenchymal tissue was collected and digested by incubation at 37 °C in TrypLE Express for approximately 10 min before being broken down into a single cell suspension by pipetting with a 200 µl pipette. The digestion was neutralized and cells were washed and pelleted before being resuspended in 950 µl freshly prepared 1% PFA (Thermo Scientific, 28908) in PBS. Cells were fixed at room temperature for 10 min while rotating. Totally, 50 µl of 2.5 M Glycine was added to quench the reaction and cells were centrifuged at 1000*g* (4 °C for 5 min). Cells were washed twice with cold PBS. After removing the PBS, cells were incubated in cold cell membrane and nuclear lysis buffers containing non-denaturing detergents for 30 min each, rotating at 4 °C. Samples were washed and pellets were suspended in a 300 µl of sonication buffer containing 0.1% SDS. Samples were sonicated in a Diagenode Bioruptor Plus (30 cycles, 30 s on, 30 s off, on high and cleared with a 1:10 volume of 10% Triton X-100. Cleared lysate was added to a mixture of protein A (Life Technologies, 10002D) and protein G beads (Life Technologies, 10004D) (20 µl each) bound to H3K27me3, H3K4me3 (EMD Millipore, CS200580), or Rabbit IgG (EMD Millipore, CS200581), and incubated overnight, rotating at 4 °C. Beads were washed 10 times with cold RIPA buffer on a magnetic rack and on ice, followed by two washes with AMBIC (100 mM). Beads were suspended in 100 µL Elution Buffer containing 2 µL of 20 mg/mL proteinase K, and incubated in a 65 °C water bath overnight. DNA was isolated by phenol/chloroform extraction and precipitated in ethanol for 2 days at −20 °C. Purified DNA was used for ChIP-qPCR analyses. Primer sequences are provided in Supplementary Table 2.

### Statistics and reproducibility

Statistical tests for significance were described in individual figure legends. All results were repeated by at least three biological replicates unless specified in figure legends. No statistical method was used to predetermine sample size. No data were excluded from the analyses. The experiments were not randomized. Blinding was done for quantification of proliferation (Fig. 2e, Fig. 4d, Fig. S9b), OLFM4 staining (Fig. 6d), TUNEL (Fig. S4b) and crypt length (Fig. 6c).

### Reporting summary

Further information on research design is available in the Nature Portfolio Reporting Summary linked to this article.

## Supplementary information

Supplementary information

Description of additional Supplementary File

Supplementary Data 1

Supplementary Data 2

Supplementari Data 3

Reporting Summary

## Data Availability

All NGS data were aligned to genome build GRCm38/mm10. Transcriptome annotations were built using “GENCODE vM4 [https://www.gencodegenes.org/mouse/release_M4.html]”. Genome and transcriptome annotations were modified to include SIRV spike-in sequences and transcript models (lot 170612a). The raw and processed NGS datasets generated in this study have been deposited in the NCBI Gene Expression Omnibus under accession code “GSE147418”. This study also makes use of data published by the ENCODE consortium under the following accession codes: “ENCFF518FTX”, “ENCFF886OPQ”, “ENCFF039QOO”, “ENCFF396TSB”, “ENCFF270YCY”, “ENCFF893IAL”, “ENCFF036PPT”, “ENCFF069OMF”, “ENCFF251XZW”, “ENCFF803SVJ”, “ENCFF829GXB”, “ENCFF280VOA”, “ENCFF180VGZ”, “ENCFF309WXH”, “ENCFF755JAU”, “ENCFF525EWD”, “ENCFF029WVD”, “ENCFF793VQQ”, “ENCFF878VPM”, “ENCFF569KWB”, “ENCFF268PNY”, “ENCFF854JVF”, “ENCFF956QXI”, “ENCFF645FMD”, “ENCFF719SDJ”, “ENCFF291JVZ”, “ENCFF902VXG”, “ENCFF445BPN”, “ENCFF882VQM”, “ENCFF554QOA”, “ENCFF548CJS”, “ENCFF027XQM”, “ENCFF491PKK”, “ENCFF904ZZH”. All other relevant data supporting the key findings of this study are available within the article and its Supplementary Information files or from the corresponding author upon reasonable request. Source data are provided with this paper.

## Source data

Source Data
