# Supplementary material for: Epigenetic control of cellular crosstalk defines gastrointestinal organ fate and function
Source: Nat Commun. 2023 Jan 30;14:497. doi: 10.1038/s41467-023-36228-2 (PMC9887003; doi:10.1038/s41467-023-36228-2)
Supplement: Supplementary file 1 — Supplementary information [file 41467_2023_36228_MOESM1_ESM.pdf]

Supplementary Information

Epigenetic control of cellular crosstalk defines gastrointestinal organ fate and function

Ryan J. Smith[1,2,*], Minggao Liang[2,3,*], Adrian Kwan Ho Loe[1,2], Theodora Yung[1,2], Ji-Eun Kim[1,2], Matthew Hudson[2,3], Michael D. Wilson[2,3], Tae-Hee Kim[1,2,‡]

[1]Program in Developmental & Stem Cell Biology, The Hospital for Sick Children, Toronto, Ontario M5G 0A4, Canada; [2]Department of Molecular Genetics, University of Toronto, Toronto, Ontario M5S 1A8, Canada; [3]Program in Genetics and Genome Biology, The Hospital for Sick Children, Toronto, Ontario M5G 0A4, Canada

* These authors contributed equally.

Keywords: Wnt signaling; stem cell niche; gastrointestinal specification; epigenetics

‡Corresponding author:

Tae-Hee Kim, PhD

The Hospital for Sick Children

Toronto, ON M5G 0A4

Tel.: 1 (416) 813-8138

Fax: 1 (416) 813-5252

Email: tae-hee.kim@sickkids.ca

This file includes Supplementary Figures (1 to 10), Supplementary Tables (1 to 2) and Supplementary Data (1 to 3) Legends.

Supplementary Figure 1

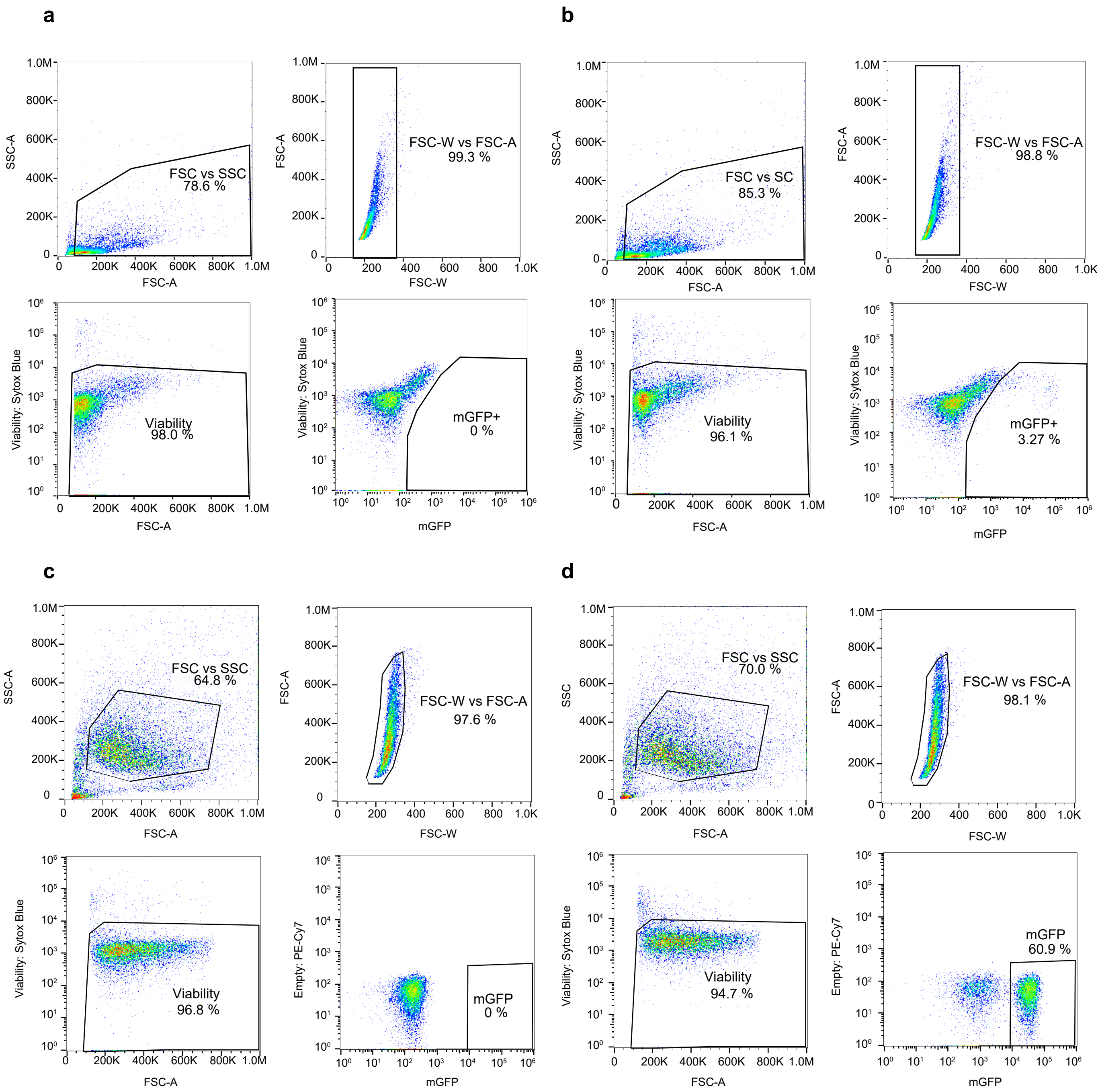

Supplementary Figure 1. Gating strategy for embryonic samples. a-b Gates used in sorting CRE- controls (**a**), and *Pdgfrβ*$^{Cre/+}$;*Rosa26*^{mTmG} and *Pdgfrβ*$^{Cre/+}$;*Eed* $^{fl/fl}$;*Rosa26*^{mTmG} mice (**b**). **c-d** Gates used in sorting GFP+ cells from CRE- controls (**c**), and *Bapx1*$^{Cre/+}$;*Rosa26*^{mTmG} and *Bapx1*$^{Cre/+}$;*Eed*$^{fl/fl}$;*Rosa26*^{mTmG} mice (**d**).

Supplementary Figure 2

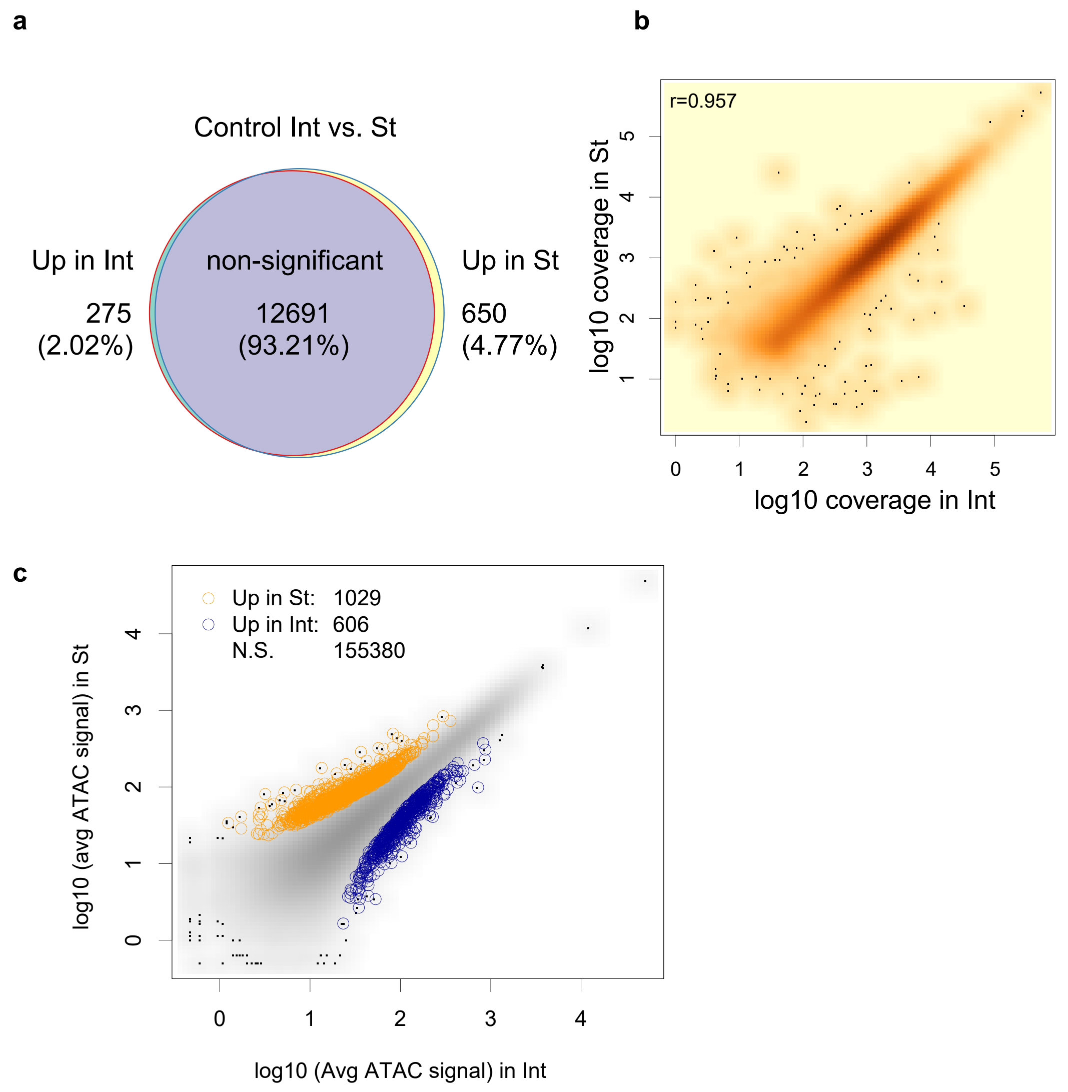

Supplementary Figure 2. Gene expression is highly similar between stomach and intestinal mesenchyme. a Overview of the number of differentially expressed genes (DEGs)identified between stomach and intestinal mesenchyme. **b** Scatterplot of log-scaled gene-level counts (averaged between replicates). Pearson's R is shown. **c** Pairwise comparison of log10-scaled ATAC-seq coverage at peaks in stomach and intestine. Significant differentially accessible regions (DARs)are marked in yellow (increased accessibility in St) and blue (increased accessibility in Int). Numbers of DARs per category are reported.

Supplementary Figure 3

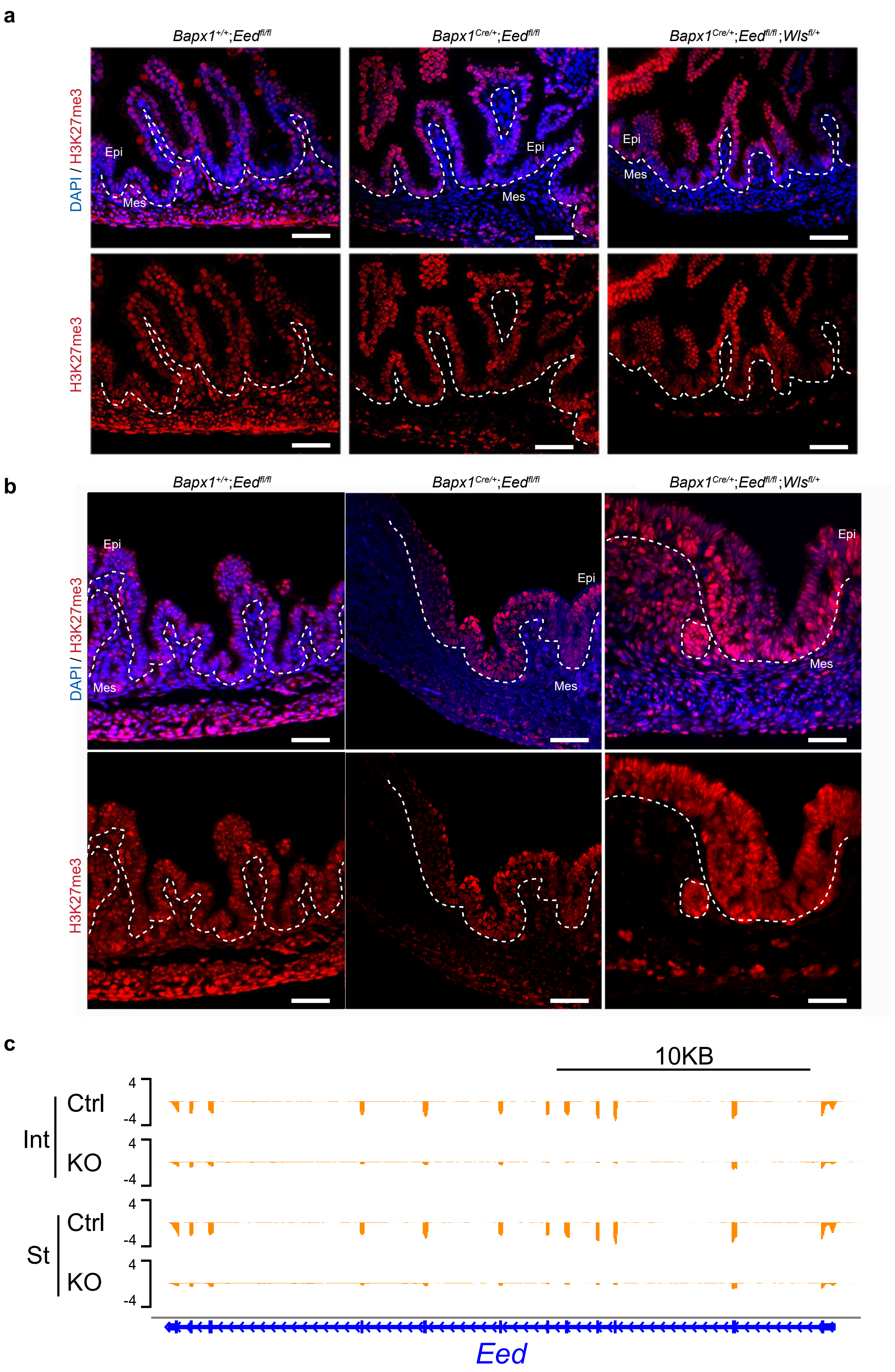

Supplementary Figure 3. Mesenchymal ablation of PRC2 activity. a-b Immunofluorescence staining of H3K27me3 demonstrates a loss of H3K27me3 in mesenchymal cells in *Bapx1*$^{Cre/+}$;*Eed*$^{fl/fl}$ and *Bapx1*$^{Cre/+}$;*Eed*$^{fl/fl}$;*Wls*$^{fl/+}$ in E17.5 intestine (**a**) and stomach (**b**). Epi – epithelium (above dashed line), Mes – mesenchyme (below dashed line), scale bars = 50µm (*n* = 3 biologically independent samples for each genotype). **c** WashU genome browser screenshot showing RNA-seq signals (merged replicates) from WT and *Eed* KO E13.5 stomach and intestinal mesenchyme. Negative values indicate reverse-stranded signals.

Supplementary Figure 4

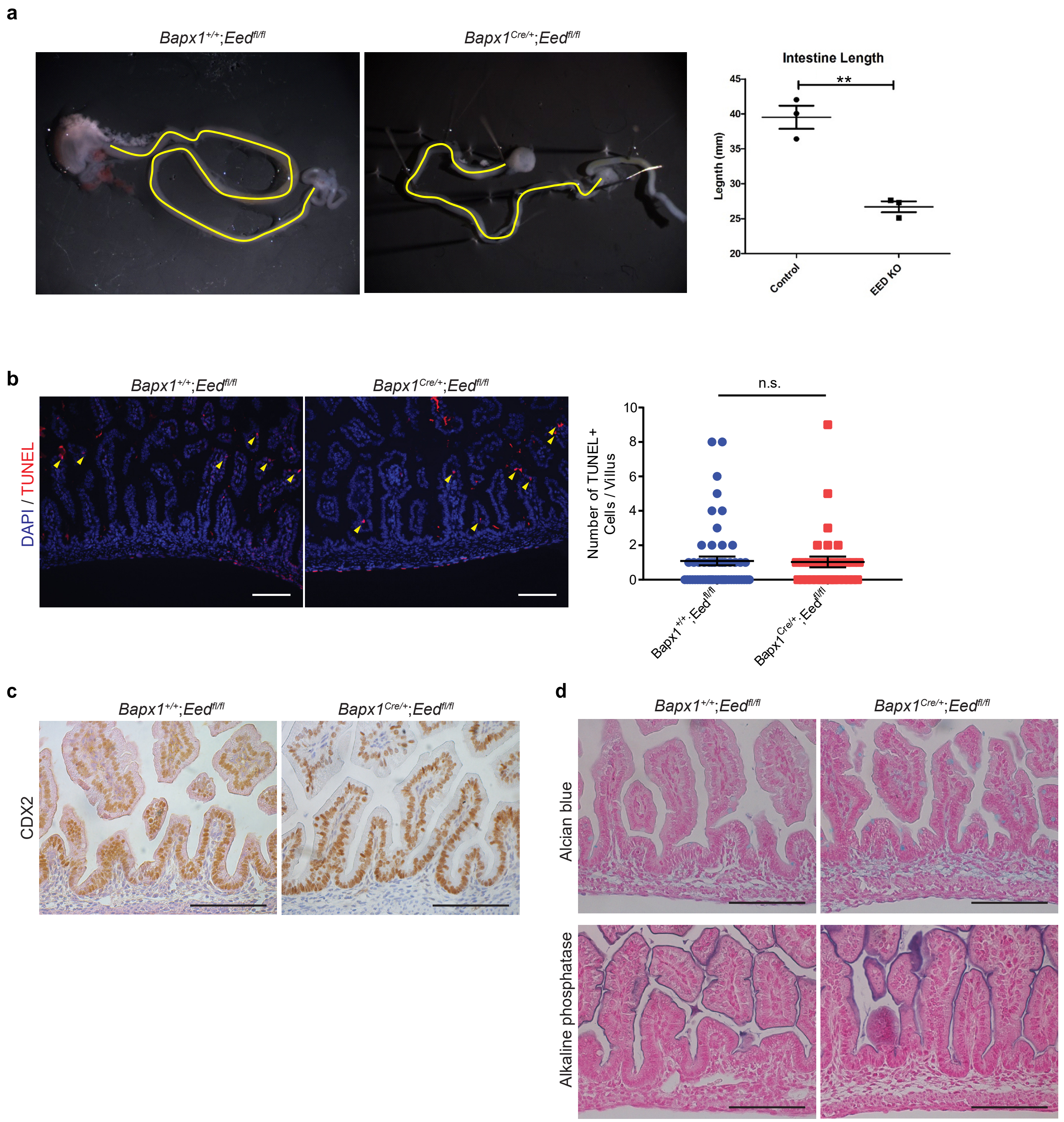

Supplementary Figure 4. Characterization of gut phenotypes upon mesenchymal PRC2 loss. a Whole mount imaging of the stomachs and intestines of *Eed* KO (*Bapx1*$^{Cre/+}$;*Eed*$^{fl/fl}$) and control mice (*Bapx1*$^{+/+}$;*Eed*$^{fl/fl}$) at E17.5. Measurement of intestinal length across *n* = 3 mice (± SEM, $**P < 0.01$ by Student's t-test, two-tailed). Source data are provided as a source data file. **b** TUNEL immunofluorescence staining shows similar numbers of apoptotic cells in the intestine of both *Eed* KO and control mice (duodenal tissues, ± SEM, *n* = 3 biologically distinct mice per genotype, n.s – Not Significant, by Student's t-test, two-tailed). Source data are provided as a source data file. **c** Immunohistochemistry against CDX2 shows its strong expression in the intestinal epithelium of both *Eed* KO and control mice (duodenal tissues; *n* = 3 biologically independent samples for each genotype). **d** Alcian blue (top) and alkaline phosphatase (bottom) staining shows the presence of intestinal goblet cells and enterocytes in both *Eed* KO and control mice (duodenal tissues; *n* = 3 biologically independent samples for each genotype).

Supplementary Figure 5

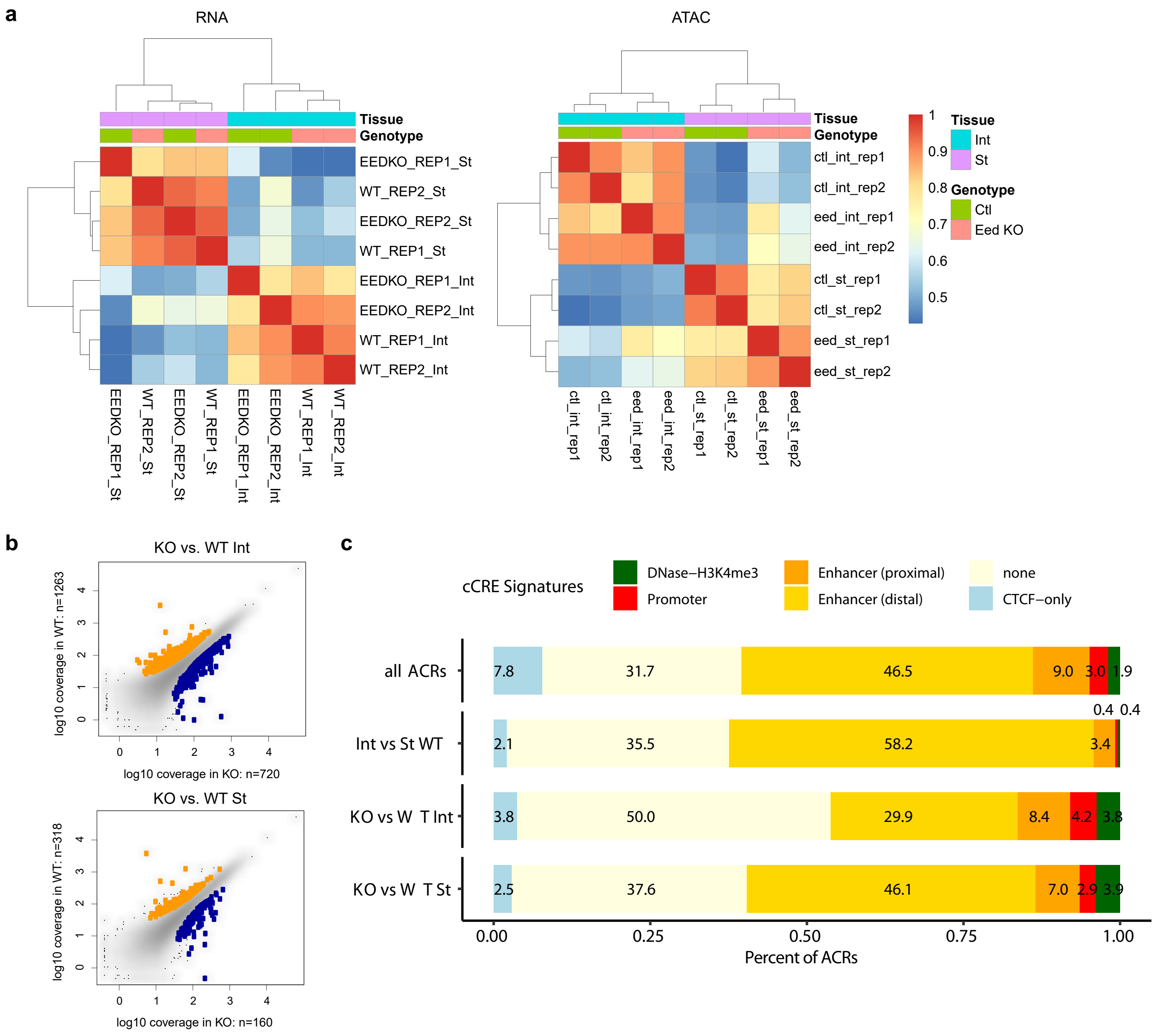

Supplementary Figure 5. Gene expression and chromatin accessibility analyses of *Eed*-knockout mesenchymal cells. a Unsupervised clustering of samples based on pairwise Pearson correlation coefficient calculated for the top 5000 most variable genes (left) and accessible chromatin regions (right). Color bars label samples by tissue and genotype. **b** Comparison of ATAC signal at accessible chromatin regions between *Eed*-KO Int (left) and St (right). DARs are marked in orange (increase in accessibility) or blue (decrease in accessibility). **c** Annotation of accessible chromatin regions (ACRs) and differentially accessible regions (DARs) using mouse pan-tissue ENCODE cCREs (accession: ENCFF904ZZH). ACRs and DARs were assigned an annotation according to the nearest cCRE within 250bp. Top row shows all ACRs across the entire dataset. Bottom rows show DARs from individual contrasts (y-axis). Values (x-axis) shows percentage calculated for total number of regions in each row.

Supplementary Figure 6

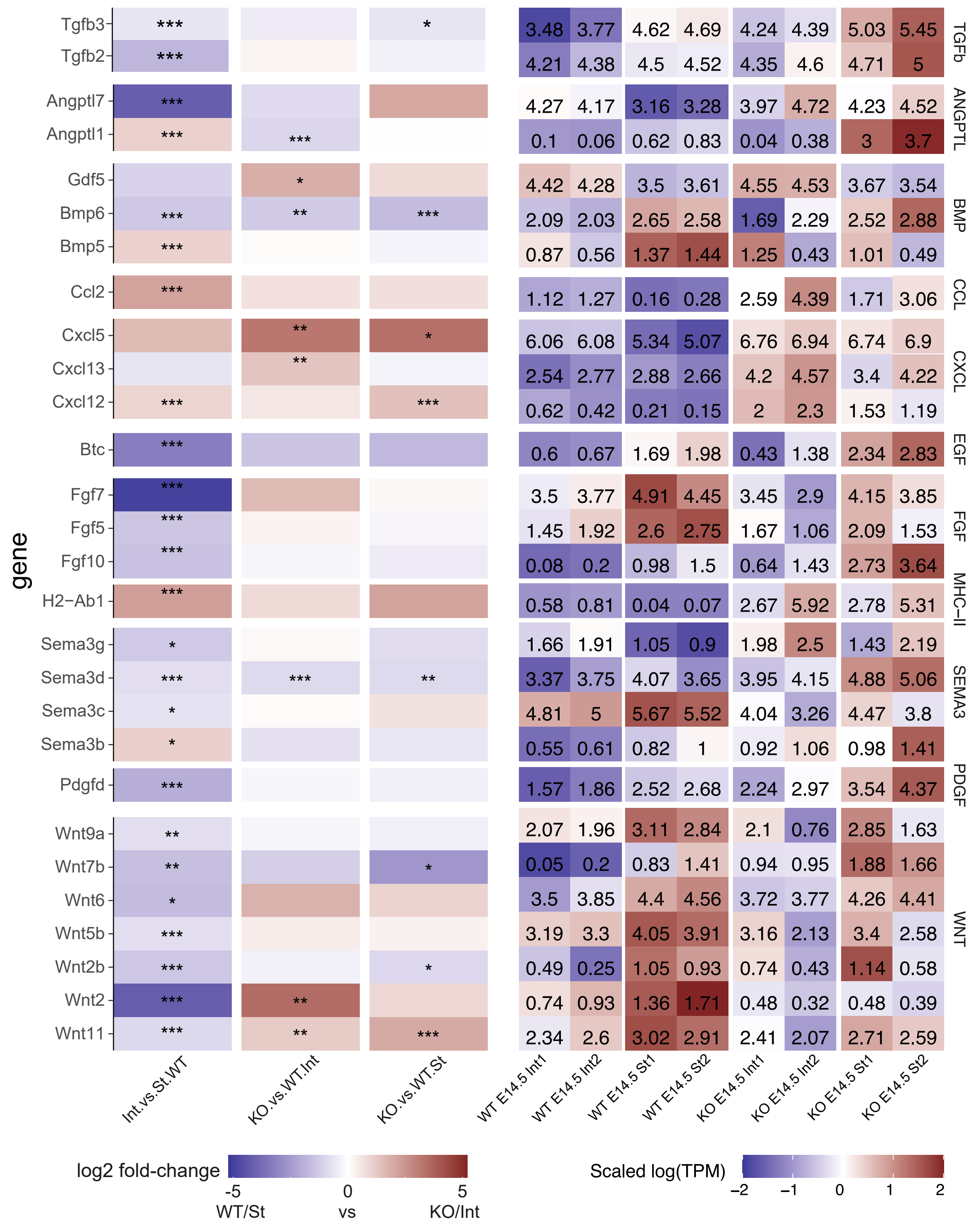

Supplementary Figure 6. Differential expression of select secreted ligands in embryonic mesenchymal cells. Comparisons between WT tissues, and between WT and *Eed* KO within tissues, are shown. Heatmap showing the TPM expression value of PRC2-sensitive ligands for all RNA-seq samples. Values show log(TPM + 1), and color shows the Z-normalized expression per gene. TPM values were calculated from spike-in normalized counts.

Supplementary Figure 7

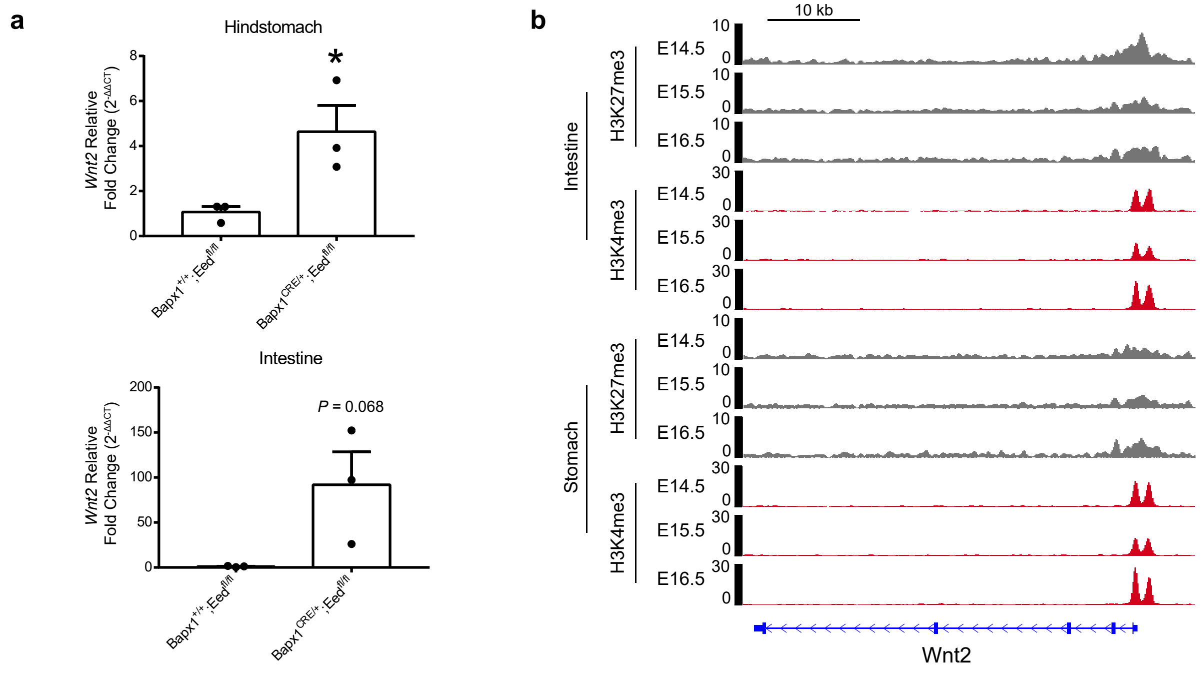

Supplementary Figure 7. Analysis of *Wnt2* expression and histone modification. a qRT-PCR of *Wnt2* in E16.5 in mesenchymal cells of control and PRC2-knockout mice (*n* = 3 biologically distinct mice per genotype, ± SEM,*$P < 0.05$, by Student's t-test, two-tailed). Source data are provided as a source data file. **b** Alignment of publicly available (see Methods) H3K27me3 and H3K4me3 ChIP-seq data at *Wnt2* to assess chromatin bivalency in E14.5, E15.5, and E16.5 whole intestine and stomach.

Supplementary Figure 8

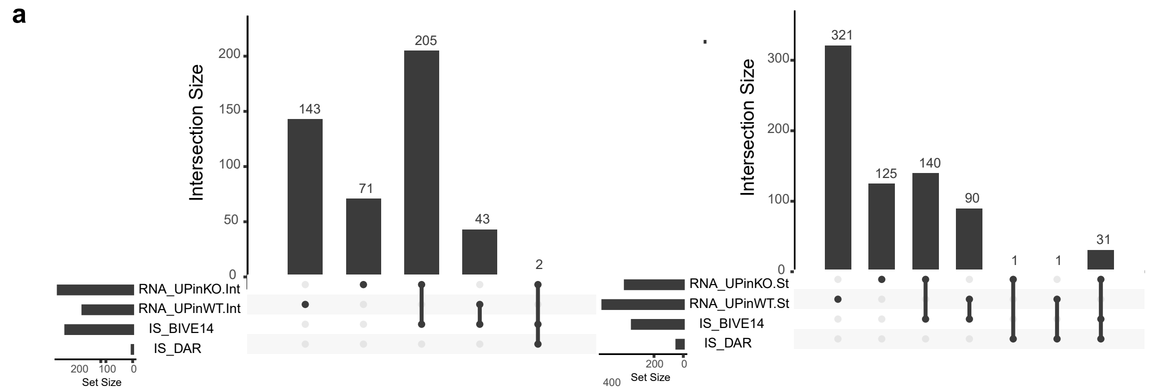

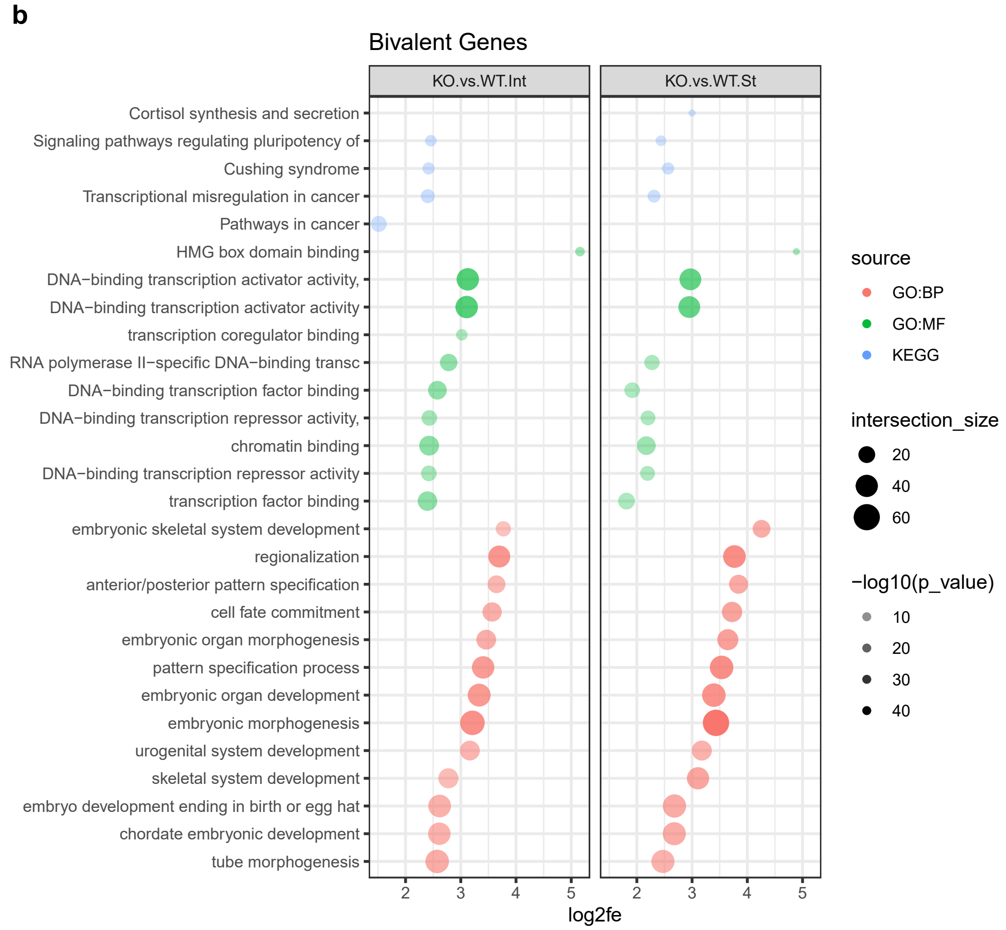

Supplementary Figure 8. Analysis of bivalent PRC2-sensitive genes. a Stratification of PRC2-sensitive genes in stomach and intestine based on promoter chromatin accessibility change (IS_DAR) and overlap with bivalent chromatin marks in at least one of stomach or intestine (IS_BIVE14). **b** GO enrichment analysis of bivalent PRC2-sensitive genes in intestine (left) and stomach (right), designated as described above. GO enrichments were performed with gProfiler2 (build:e105_eg52_p16_e84549f) in R. Only the top 10 significant hits (P<0.01), ranked by fold-enrichment per tissue and per ontology source, are shown.

Supplementary Figure 9

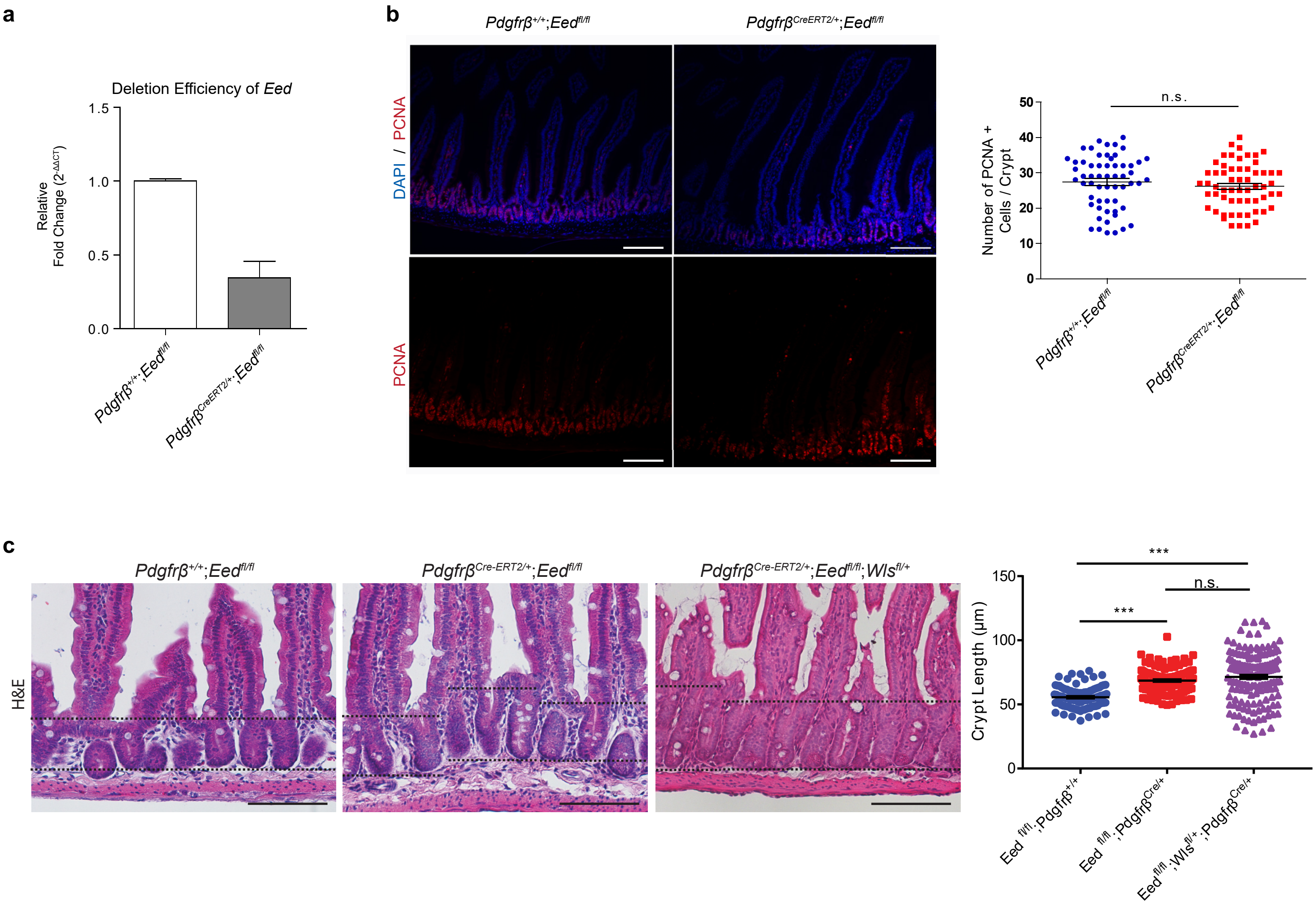

Supplementary Figure 9. PRC deletion in pericryptal cells does not alter proliferation. **a** Deletion efficiency of *Eed* in pericryptal cells isolated from *Pdgfrβ*$^{Cre/+}$;*Eed* $^{fl/fl}$;*Rosa26*^{mTmG} and *Pdgfrβ*$^{+/+}$;*Eed* $^{fl/fl}$;*Rosa26*^{mTmG} mice (*n* = 2 biologically independent animals, mean ± SEM). Source data are provided as a source data file. **b** Immunofluorescence staining of PCNA. Number of PCNA positive cells per crypt were quantified in *n* = 3 biologically distinct mice per genotype (± SEM, n.s – Not Significantm by Student's t-test, two-tailed). Each data point represents an independent crypt measurement. Source data are provided as a source data file. **c** Haematoxylin and Eosin staining of the intestines of *Pdgfrβ*$^{Cre/+}$;*Eed*$^{fl/fl}$, *Pdgfrβ*$^{Cre/+}$;*Eed*$^{fl/fl}$, and *Pdgfrβ*$^{Cre/+}$;*Eed*$^{fl/fl}$;*Wls*$^{fl/+}$ mice shows an increased crypt length in *Eed* KOs compared to controls, while *Wls* heterozygosity causes no further changes in crypt length (*n* = 3 biologically independent animals, mean ± SEM, *** p<0.001, n.s – Not Significant, by Student's t-test, two-tailed). Source data are provided as a source data file. Scale bars = 100µm.

Supplementary Figure 10

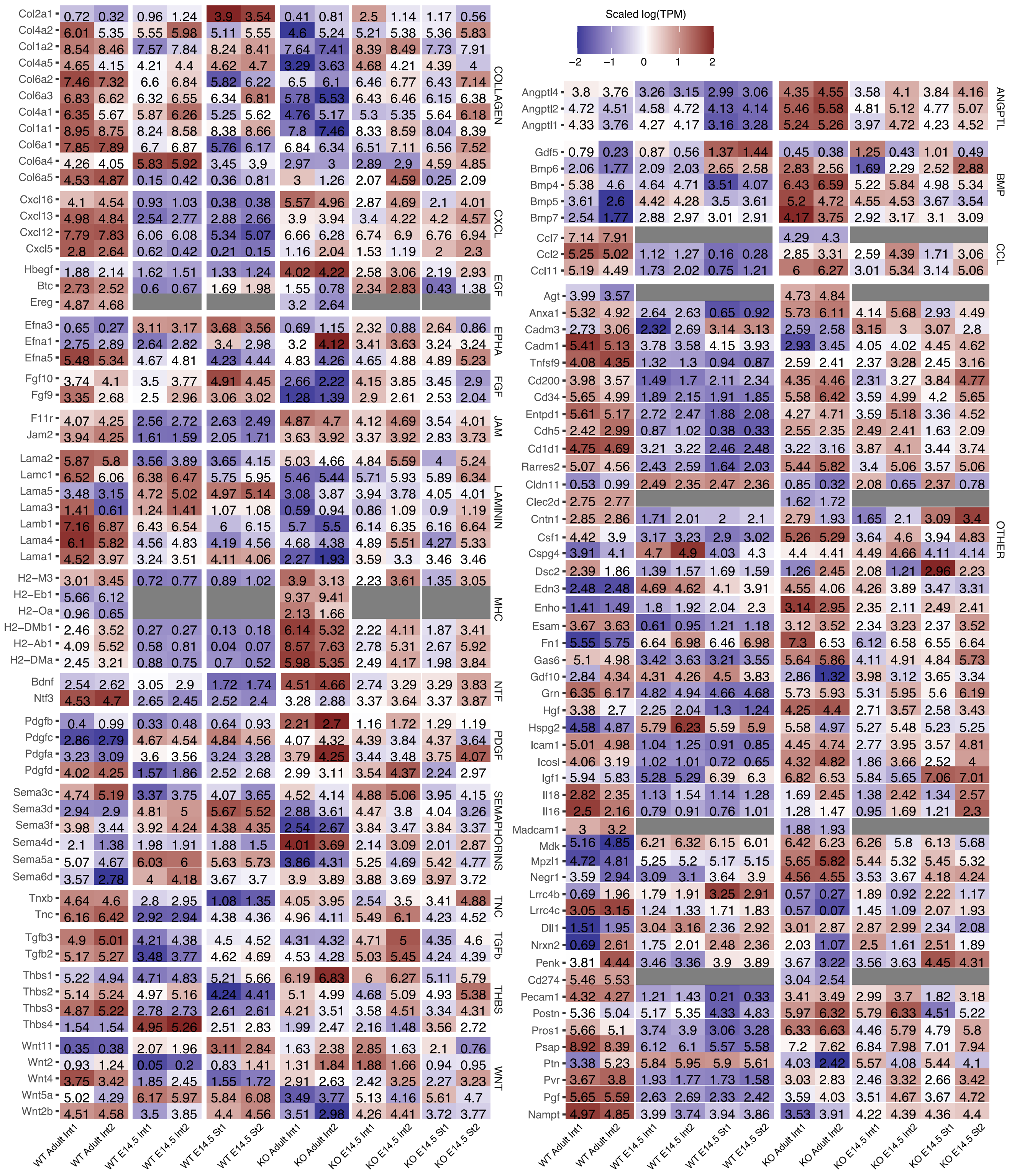

Supplementary Figure 10. Differential expression of secreted ligands in adult and embryonic intestinal mesenchymal cells upon *Eed* KO. Heatmap showing the TPM expression value of PRC2-sensitive ligands for all RNA-seq samples. Values show log(TPM + 1), while color represents the Z-normalized expression per gene. TPM values were calculated from spike-in normalized counts.

Supplementary Table 1. Datasets from the ENCODE consortium used in this study.

File Type	Organ	Histone Mark	Stage	ENCODE ascension codes
bigWig	Stomach	H3K27me3	E14.5	ENCFF518FTX
			E15.5	ENCFF886OPQ
			E16.5	ENCFF039QOO
	Intestine	H3K27me3	E14.5	ENCFF396TSB
			E15.5	ENCFF270YCY
			E16.5	ENCFF893IAL
	Stomach	H3K27ac	E14.5	ENCFF036PPT
			E16.5	ENCFF069OMF
	Intestine	H3K27ac	E14.5	ENCFF251XZW
			E15.5	ENCFF803SVJ
			E16.5	ENCFF829GXB
	Stomach	H3K4me3	E14.5	ENCFF280VOA
			E15.5	ENCFF180VGZ
			E16.5	ENCFF309WXH
	Intestine	H3K4me3	E14.5	ENCFF755JAU
			E15.5	ENCFF525EWD
			E16.5	ENCFF029WVD
bed (narrow Peak)	Stomach	H3K4me3	E14.5	ENCFF793VQQ
			E15.5	ENCFF878VPM
			E16.5	ENCFF569KWB
			P0	ENCFF268PNY
	Intestine	H3K4me3	E14.5	ENCFF854JVF
			E15.5	ENCFF956QXI
			E16.5	ENCFF645FMD
			P0	ENCFF719SDJ
ChromHMM (bed9)				ENCFF291JVZ
				ENCFF902VXG
				ENCFF445BPN
				ENCFF882VQM
				ENCFF554QOA
				ENCFF548CJS
				ENCFF027XQM
				ENCFF491PKK

Supplementary Table 2. Primer sequences used in this study.

**Gene/locus**	**Purpose**	**Sequence**
Neg	ChIP-qPCR	Forward: 5’ – ACAACATCCACACGTCCAGTGATT – 3’<br>Reverse: 5’ – ACAACATCCACACGTCCAGTGATT – 3’
Gapdh	ChIP-qPCR	Forward: 5’ – TATGCCCGAGGACAATAAGG – 3’<br>Reverse: 5’ – CGCCGTTATGAAATCTTGCT – 3’
Actb	ChIP-qPCR	Forward: 5’ – CGTAGGCCCAGATGTACAGG – 3’<br>Reverse: 5’ – CAAACAAGAGGCCACACAAA – 3’
Hoxd12	ChIP-qPCR	Forward: 5’ – AATTGGTGGCCGTAACTCAC – 3’<br>Reverse: 5’ – GCCAAGCTGGAACTGGTTAG – 3’
Wnt2	ChIP-qPCR	Forward: 5’ – CTTTCCGTTCCCCTCCAG – 3’<br>Reverse: 5’ – GGAGATAGCTCTTGCCATCG – 3’
Gapdh	RT-qPCR	Forward: 5’ – TCGTCCCGTAGACAAAATGG – 3’<br>Reverse: 5’ – GAGGTCAATGAAGGGGTCGT – 3’
Wnt2	RT-qPCR	Forward: 5’ – CTCGGTGGAATCTGGCTCTG – 3’<br>Reverse: 5’ – CACATTGTCACACATCACCCT – 3’
Eed	RT-qPCR	Forward: 5’ – GTGTGGAAGGGCACAGAGAT – 3’<br>Reverse: 5’ – ATCAAATCGCCTAACCATCG – 3’

Supplementary Data 1. DESEQ2 results of differentially expressed genes identified between organs and between control and *Eed* KO conditions, as determined by RNA-seq.

Supplementary Data 2. DESEQ2 results of differentially accessible regions identified between organs and between control and *Eed* KO conditions, as determined by ATAC-seq.

Supplementary Data 3. Joint DESEQ2 results, which combine the DESEQ2 results from RNA-seq and ATAC-seq.
