# Supplementary material for: Epigenetic control of cellular crosstalk defines gastrointestinal organ fate and function
Source: Nat Commun. 2023 Jan 30;14:497. doi: 10.1038/s41467-023-36228-2 (PMC9887003; doi:10.1038/s41467-023-36228-2)
Supplement: Supplementary file 2 — Description of additional Supplementary File [file 41467_2023_36228_MOESM2_ESM.pdf]

Descriptions of additional supplementary files

Supplementary Data 1. DESEQ2 results of differentially expressed genes identified between organs and between control and Eed KO conditions, as determined by RNA-seq.

Supplementary Data 2. DESEQ2 results of differentially accessible regions identified between organs and between control and Eed KO conditions, as determined by ATAC-seq.

Supplementary Data 3. Joint DESEQ2 results, which combine the DESEQ2 results from RNA-seq and ATAC-seq.
