# Supplementary material for: Epigenetic control of cellular crosstalk defines gastrointestinal organ fate and function
Source: Nat Commun. 2023 Jan 30;14:497. doi: 10.1038/s41467-023-36228-2 (PMC9887003; doi:10.1038/s41467-023-36228-2)
Supplement: Supplementary file 6 — Reporting Summary [file 41467_2023_36228_MOESM6_ESM.pdf]

nature portfolio

Corresponding author(s): Tae-Hee Kim
Last updated by author(s): Dec 27, 2022

Reporting Summary

Nature Portfolio wishes to improve the reproducibility of the work that we publish. This form provides structure for consistency and transparency in reporting. For further information on Nature Portfolio policies, see our Editorial Policies and the Editorial Policy Checklist.

Statistics

For all statistical analyses, confirm that the following items are present in the figure legend, table legend, main text, or Methods section.

n/a	Confirmed	
☐	☒	The exact sample size (*n*) for each experimental group/condition, given as a discrete number and unit of measurement
☐	☒	A statement on whether measurements were taken from distinct samples or whether the same sample was measured repeatedly
☐	☒	The statistical test(s) used AND whether they are one- or two-sided<br>*Only common tests should be described solely by name; describe more complex techniques in the Methods section.*
☐	☒	A description of all covariates tested
☐	☒	A description of any assumptions or corrections, such as tests of normality and adjustment for multiple comparisons
☐	☒	A full description of the statistical parameters including central tendency (e.g. means) or other basic estimates (e.g. regression coefficient) AND variation (e.g. standard deviation) or associated estimates of uncertainty (e.g. confidence intervals)
☐	☒	For null hypothesis testing, the test statistic (e.g. F, t, r) with confidence intervals, effect sizes, degrees of freedom and P value noted<br>*Give P values as exact values whenever suitable.*
☒	☐	For Bayesian analysis, information on the choice of priors and Markov chain Monte Carlo settings
☐	☒	For hierarchical and complex designs, identification of the appropriate level for tests and full reporting of outcomes
☐	☒	Estimates of effect sizes (e.g. Cohen's *d*, Pearson's *r*), indicating how they were calculated

Our web collection on statistics for biologists contains articles on many of the points above.

Software and code

Policy information about availability of computer code

Data collection	Illumina base-calling: bcl2fastq2 v2.20
Data analysis	Genome Alignment (ATAC-seq): bwa v0.7.8<br>Genome Alignment (RNA-seq): STAR v2.5.1b<br>Read counting: featureCounts v1.5.0<br>Peak Calling: MACS v2.1.2<br>Differential Enrichment Analysis: DESeq2 v1.24<br>GraphPad Prism 7 software was used for quantifying counts.<br>The "Find Maxia" function of FIJI (version 1.52r) was used to quantify Axin2 expression<br>Cytobank 7.2

For manuscripts utilizing custom algorithms or software that are central to the research but not yet described in published literature, software must be made available to editors and reviewers. We strongly encourage code deposition in a community repository (e.g. GitHub). See the Nature Portfolio guidelines for submitting code & software for further information.

Data

Policy information about availability of data

All manuscripts must include a data availability statement. This statement should provide the following information, where applicable:

- Accession codes, unique identifiers, or web links for publicly available datasets
- A description of any restrictions on data availability
- For clinical datasets or third party data, please ensure that the statement adheres to our policy

All NGS data were aligned to genome build GRCm38/mm10. Transcriptome annotations were built using GENCODE vM4 https://www.gencodegenes.org/mouse/release_M4.html. Genome and transcriptome annotations were modified to include SIRV spike-in sequences and transcript models (lot 170612a) https://www.lexogen.com/wp-content/uploads/2021/06/Previous_versions_SIRV-Set_3_until_2020.zip. Raw and processed NGS datasets are available through GEO accession GSE147418 https://www.ncbi.nlm.nih.gov/geo/query/acc.cgi?acc=GSE147418.

This study makes use of data published by the ENCODE consortium, under the following accession codes:
ENCFF518FTX: https://www.encodeproject.org/files/ENCFF518FTX/
ENCFF886OPQ: https://www.encodeproject.org/files/ENCFF886OPQ/
ENCFF039QOO: https://www.encodeproject.org/files/ENCFF039QOO/
ENCFF396TSB: https://www.encodeproject.org/files/ENCFF396TSB/
ENCFF270YCY: https://www.encodeproject.org/files/ENCFF270YCY/
ENCFF893IAL: https://www.encodeproject.org/files/ENCFF893IAL/
ENCFF036PPT: https://www.encodeproject.org/files/ENCFF036PPT/
ENCFF069OMF: https://www.encodeproject.org/files/ENCFF069OMF/
ENCFF251XZW: https://www.encodeproject.org/files/ENCFF251XZW/
ENCFF803SVJ: https://www.encodeproject.org/files/ENCFF803SVJ/
ENCFF829GXB: https://www.encodeproject.org/files/ENCFF829GXB/
ENCFF280VOA: https://www.encodeproject.org/files/ENCFF280VOA/
ENCFF180VGZ: https://www.encodeproject.org/files/ENCFF180VGZ/
ENCFF309WXH: https://www.encodeproject.org/files/ENCFF309WXH/
ENCFF755JAU: https://www.encodeproject.org/files/ENCFF755JAU/
ENCFF525EWD: https://www.encodeproject.org/files/ENCFF525EWD/
ENCFF029WVD: https://www.encodeproject.org/files/ENCFF029WVD/
ENCFF793VQQ: https://www.encodeproject.org/files/ENCFF793VQQ/
ENCFF878VPM: https://www.encodeproject.org/files/ENCFF878VPM/
ENCFF569KWB: https://www.encodeproject.org/files/ENCFF569KWB/
ENCFF268PNY: https://www.encodeproject.org/files/ENCFF268PNY/
ENCFF854JVF: https://www.encodeproject.org/files/ENCFF854JVF/
ENCFF956QXI: https://www.encodeproject.org/files/ENCFF956QXI/
ENCFF645FMD: https://www.encodeproject.org/files/ENCFF645FMD/
ENCFF719SDJ: https://www.encodeproject.org/files/ENCFF719SDJ/
ENCFF291JVZ: https://www.encodeproject.org/files/ENCFF291JVZ/
ENCFF902VXG: https://www.encodeproject.org/files/ENCFF902VXG/
ENCFF445BPN: https://www.encodeproject.org/files/ENCFF445BPN/
ENCFF882VQM: https://www.encodeproject.org/files/ENCFF882VQM/
ENCFF554QOA: https://www.encodeproject.org/files/ENCFF554QOA/
ENCFF548CJS: https://www.encodeproject.org/files/ENCFF548CJS/
ENCFF027XQM: https://www.encodeproject.org/files/ENCFF027XQM/
ENCFF491PKK: https://www.encodeproject.org/files/ENCFF491PKK/

Human research participants

Policy information about studies involving human research participants and Sex and Gender in Research.

Reporting on sex and gender	No human participants are involved.
Population characteristics	*Describe the covariate-relevant population characteristics of the human research participants (e.g. age, genotypic information, past and current diagnosis and treatment categories). If you filled out the behavioural & social sciences study design questions and have nothing to add here, write "See above."*
Recruitment	*Describe how participants were recruited. Outline any potential self-selection bias or other biases that may be present and how these are likely to impact results.*
Ethics oversight	*Identify the organization(s) that approved the study protocol.*

Note that full information on the approval of the study protocol must also be provided in the manuscript.

Field-specific reporting

Please select the one below that is the best fit for your research. If you are not sure, read the appropriate sections before making your selection.

- [x] Life sciences
- [ ] Behavioural & social sciences
- [ ] Ecological, evolutionary & environmental sciences

For a reference copy of the document with all sections, see nature.com/documents/nr-reporting-summary-flat.pdf

Life sciences study design

All studies must disclose on these points even when the disclosure is negative.

Sample size	n = 2 biological replicates are used for sequencing experiments. A minimum of n = 3 biologically independent samples were used in all image quantification. No statistical method was used to predetermine sample size. For mutant mouse strains, difficulties of acquiring mutant genotype limit sample size.
Data exclusions	No data were exluded.
Replication	All phenotypes are reproduced consistently in the indicated n number of biological distinct animals.
Randomization	Allocation into groups was determined by genotype after dissection.
Blinding	Blinding was done for quantification of proliferation (Fig 2. E, Fig 4 D, Supple. Fig 9 B), OLFM4 staining (Fig. 6 D), TUNEL (Supple. Fig. 4 B) and crypt length (Fig. 6 C). Blinding was not necessary for sequencing experiments and imaging, as no data were excluded.

Reporting for specific materials, systems and methods

We require information from authors about some types of materials, experimental systems and methods used in many studies. Here, indicate whether each material, system or method listed is relevant to your study. If you are not sure if a list item applies to your research, read the appropriate section before selecting a response.

Materials & experimental systems

n/a	Involved in the study	
☐	☒	Antibodies
☒	☐	Eukaryotic cell lines
☒	☐	Palaeontology and archaeology
☐	☒	Animals and other organisms
☒	☐	Clinical data
☒	☐	Dual use research of concern

Methods

n/a	Involved in the study	
☒	☐	ChIP-seq
☐	☒	Flow cytometry
☒	☐	MRI-based neuroimaging

Antibodies

Antibodies used

For immunofluorescence staining:
Mouse anti-PCNA 1:200 (Santa Cruz Biotechnology, sc-56, lot C0816)
Rabbit anti-H3K27me3, 1:750 (EMD Millipore, 07-449)
Rabbit anti-OLFM4, 1:100 (Cell Signaling Technology, D6Y5A)
Mouse anti-GFP, 1:300 (1:300; Santa Cruz Biotechnology, 9996)
Goat anti-mouse alexa fluor 568, 1:500 (Thermo Fisher Scientific, A11031)
Goat anti-rabbit alexa fluor 568, 1:500 (Thermo Fisher Scientific, A11036)
Goat anti-mouse alexa fluor 488, 1:500 (Thermo Fisher Scientific, A11029)

For Immunohistochemistry staining:
Mouse anti-PDX1 1:300 (Developmental Studies Hybridoma Bank, F109-D12)
Mouse anti-CDX2, 1:300 (Biogenex, MU392A-UC)
Biotin-conjugated anti-mouse, 1:200 (Vector Laboratories, BA-9200)

For Chromatin Immunoprecipitation:
Rabbit anti-H3K27me3, 1ug per 10uL of beads used (EMD Millipore, 07-449)
Rabbit anti-H3K4me3, 1ug per 10uL of beads used (EMD Millipore, CS200580)
Rabbit anti-IgG, 1 ug per 10uL of beads used (EMD Millipore, CS200581)

Validation

For immunofluorescence staining:
Mouse anti-PCNA 1:200 (Santa Cruz Biotechnology, sc-56, lot C0816)
reactivity: mouse, rat, human
application: WB, IP, ICC, IHC, FACS
commercial test: ICC on HeLa, IHC on human lymph node tissue, WB on HCT-166/Raji/HeLa/MOLT-4/NIH/3T3/KNRK

Rabbit anti-H3K27me3, 1:750 (EMD Millipore, 07-449)
reactivity: human, mouse
application: ICC, IP, Mplex, WB, IHC
commercial test: ICC on MEF, WB on HeLa, WB on peptides

Rabbit anti-OLFM4, 1:100 (Cell Signaling Technology, D6Y5A)
reactivity: mouse
application: WB, IP, IHC, ICC
commercial test: WB onmouse intestine/spleen/colon, IHC on mouse intestinal adenoma, IHC on mouse small intestine and colon, IHC on mouse spleen, IF on mouse small intestine

Mouse anti-GFP, 1:300 (1:300; Santa Cruz Biotechnology, 9996)
reactivity: not applicable
application: WB, IP, IF, FCM, ELISA
commercial test: ICC on COS, WB on COS, WB on recombinant protein

Mouse anti-PDX1 1:300 (Developmental Studies Hybridoma Bank, F109-D12)
reactivity: mouse rat
application: ELISA, FACS, IF, IHC, WB
commercial test: no commercial test shown on manufacturer website, but applications are referenced

Mouse anti-CDX2, 1:300 (Biogenex, MU392A-UC)
reactivity: mouse, human
application: IHC, ICC
commercial test: no commercial test shown on manufacturer website, but applications are referenced

Rabbit anti-H3K4me3, 1ug per 10uL of beads used (EMD Millipore, CS200580)
reactivity: human, mouse
application: WB, ChIP, ChIP-seq
commercial test: ChIP in HeLa/U2OS

Rabbit anti-IgG, 1 ug per 10uL of beads used (EMD Millipore, CS200581)
reactivity: not applicable
application: IP, WB
commercial test: no commercial test shown on manufacturer website, but applications are referenced

Animals and other research organisms

Policy information about studies involving animals; ARRIVE guidelines recommended for reporting animal research, and Sex and Gender in Research

Laboratory animals

Male and female mice (mus musculus) from mixed strain backgrounds were harvested at E13.5, E16.5, E17.5, and approximately 10 weeks of age for this study.

Background strains of mouse lines used in this study:
PdgfrβCre-ERT2: mixed 129 × C57BL/6
Eedfl: mixed 129 × C57BL/6
Bapx1Cre: C57BL/6
Wlsfl: mixed 129 x 129S1/SvlmJ
Rosa26mTmG: mixed C57BL/6J x CD-1

Up to 5 adult mice are housed in one cage with water valve, dried food pellet and nesting. Animals are housed in The Centre for Phenogenomics located in Toronto, Ontario. Additional information regarding housing conditions can be found on The Centre for Phenogenomics website.

Wild animals

This study did not involve wild animals.

Reporting on sex

Sex was not relevant in this study.

Field-collected samples

No samples were collected from the field.

Ethics oversight	All mice were handled in accordance with the rules and regulations of the Canadian Council on Animal Care Guidelines for Use of Animals in Research and Laboratory Animal Care under protocols approved by the Animal Care Committee at The Centre for Phenogenomics (protocol: 19-0276H).

Note that full information on the approval of the study protocol must also be provided in the manuscript.

Flow Cytometry

Plots

Confirm that:

- [x] The axis labels state the marker and fluorochrome used (e.g. CD4-FITC).
- [x] The axis scales are clearly visible. Include numbers along axes only for bottom left plot of group (a 'group' is an analysis of identical markers).
- [x] All plots are contour plots with outliers or pseudocolor plots.
- [x] A numerical value for number of cells or percentage (with statistics) is provided.

Methodology

Sample preparation	For embryonic samples:<br>E13.5 GFP+ embryos were identified through fluorescence microscopy. Stomachs and intestines of each animal were separated through microdissection and placed in 2% Fetal Bovine Serum in ultra-pure PBS. Samples were then centrifuged at 4°C for 5 minutes at 400 RCF. Samples were digested in 3ml of a 2:1 ratio of TrypLE Express to ultrapure PBS until tissues clumped together (approximately 10 minutes). Tissue were then subject to manual dissociation with a p200 pipette until a single-cell solution was observed. Samples were then neutralized with and equal volume of 2% FBS in ultrapure PBS and centrifuged at the conditions described above. Samples were washed in ultrapure 2% FBS and centrifuged again. After decanting the solution, cells were resuspended in ultrapure 2% FBS containing a 1:5000 dilution of Sytox Blue, and filtered through 35µm mesh into a polypropylene tube in preparation for FACS using the Sony SH800 BRV instrument.<br><br>For adult samples:<br>Briefly, small intestines were isolated, opened, and washed in GB1 (HBSS with 10% fetal calf serum, and 10mM HEPES). Villi were carefully scraped off using a glass slide and discarded. Remaining tissue was cut into small pieces, vortexed, and washed twice in GB1. Tissues were transferred to GB2 (GB1 with 10mM EDTA) and shaken for 20min (220 RPM) at 37°C before being vortexed and washed through a 100µm to removed epithelial cells. This process was repeated twice and remaining tissues were washed in GB1 before being placed in digestion solution(20ml RPMI, 1% Penicillin-Streptomycin, 10% FBS, 15mM HEPES, 2mg DNAse I , 3.12mg Protease IV, 58mg Dispase II for 1 hour at 37°C, vortexing every 15 minutes. The cell suspension was poured through a 70µm strainer and centrifuged at 400g for 5 min at 4°C. The supernatant was removed and this wash was repeated. Cells were resuspended in 2% FBS with 1:5000 Sytox Blue and filtered through a 35 µm mesh in preparation for FACS using the Sony SH800 BRV instrument.
Instrument	The Sony SH800 BRV instrument was used.
Software	Cytobank 7.2 was used to analyze flow cytometry data.
Cell population abundance	Embryonic:<br>GFP-positive mesenchymal cells represent approximately half (40-60%) of all live cells in both stomach and intestine samples for both control and PRC2-ablated populations.<br>Purity was determined by running a negative control containing only Sytox Blue, without possessing GFP.<br><br>Adult:<br>GFP-positive cells represent about 2% of all viable cells. Purity was determined by running a negative control to establish true GFP signal.
Gating strategy	Embryonic:<br>Cells with low FSC and SSC were excluded, as this could represent debris. The largest fraction of suitably sized cells was collected, excluding larger sizes which may represent doublets or clumps of undigested cells. Cells were considered viable by Sytox Blue when they were below a set threshold, as per the manufacturer's directions. Thresholds for GFP expression were determined through the use of GFP-negative controls. Signal beyond this negative-control threshold was considered GFP-positive.<br><br>Adult:<br>The lowest FSC and SCS populations were excluded as these may represent debris. Given that PDGFRB-expressing cells are known to be large, we included much of the outlying population as well. Viability and GFP expression were determined in he same manner as embryonic tissues.

- [x] Tick this box to confirm that a figure exemplifying the gating strategy is provided in the Supplementary Information.
